# Exploring patient treatment decision making for osteoarthritis in the UAE: a cross-sectional adaptive choice-based conjoint study

**DOI:** 10.1186/s12889-023-16490-1

**Published:** 2023-08-12

**Authors:** Basem Al-Omari, Joviana Farhat, Mumtaz Khan, Hristo Grancharov, Zaki Abu Zahr, Sammy Hanna, Abdulla Alrahoomi

**Affiliations:** 1 Department of Epidemiology and Population Health, College of Medicine and Health Sciences, Khalifa University, P.O. Box 127788, Abu Dhabi, United Arab Emirates; 2 Department of Rheumatology, Sheikh Shakhbout Medical City (SSMC), P.O. Box 11001, Abu Dhabi, United Arab Emirates; 3 Department of Orthopedics & Sports Medicine, Healthpoint Hospital, P.O. Box 112308, Abu Dhabi, United Arab Emirates; 4 Department of Rheumatology, Healthpoint Hospital, P.O. Box 112308, Abu Dhabi, United Arab Emirates; 5 Barts and the London School of Medicine and Dentistry, Queen Mary University of London, Whitechapel, London, E1 2AD UK

**Keywords:** Osteoarthritis, Patients’ preferences, Adaptive choice-based conjoint, Pharmaceutical treatment, Web-based questionnaires

## Abstract

**Objective:**

To assess osteoarthritis (OA) patients’ preferences for pharmaceutical treatment via Adaptive Choice-Based Conjoint (ACBC) method.

**Methods:**

A United Arab Emirates (UAE) based Patient and Public Involvement (PPI) group designed the ACBC questionnaire with 10 attributes and 34 levels. The questionnaire was developed using Sawtooth Software and analyzed through Hierarchical Bayesian (HB). Results were standardized using Z-score via SPSS.

**Results:**

Study participants were 1030 OA patients, 83.6% aged 50 or older and 83.4% female. The avoidance of medication’s side effects accounted for 66% relative importance compared to 6% relative importance for the medication’s benefits. The “way of taking the medicine” attribute had the highest coefficient of variation (70%) and the four side effect attributes “risk of gastric ulcer, addiction, kidney and liver impairment, and heart attacks and strokes” had a coefficient of variation from 18 to 21%.

**Conclusions:**

Arab OA patients are similar to other ethnic groups in trading-off benefits and side effects and consistently prioritizing the avoidance of medications’ side effects. Although the “Way of taking medicine” was the least important attribute it was associated with the highest variation amongst patients. OA patients also prefer prescribed medications to internet-purchased and over-the-counter options.

**Supplementary Information:**

The online version contains supplementary material available at 10.1186/s12889-023-16490-1.

## Introduction

Osteoarthritis (OA) is a highly prevalent chronic joint disease, affecting 7% of the world population [1–3]. The treatment for OA includes non-pharmacological and pharmacological options, which focus on alleviating pain and stiffness and limiting functional loss [4]. Due to the long-term use of the pharmacological treatment and the adverse effects, surgical interventions such as joint replacement may be considered the last option [5, 6].

The assessment of patients’ preferences is a crucial aspect of healthcare systems worldwide [7]. Especially, in the management of non-urgent and non-fatal diseases, such as OA [8]. Appropriate elicitation of patients’ preferences for treatment and involving patients in the shared decision-making (SDM) process can improve disease management and patient adherence [9]. Furthermore, understanding patients’ preferences can aid in balancing the treatment’s benefits and risks [10, 11], and identifying choices of subpopulations with different risk tolerances [12]. Subsequently, the new treatment design and prescription could focus on those attributes that are highly important to the patients.

In Arabic-speaking countries, the burden of chronic diseases is increasing, but the consideration of patients’ opinions regarding treatment options is limited [13, 14]. This leads to the under-representation of patients’ needs and preferences in SDM [15, 16]. While studying patients’ preferences and SDM have been widely implemented in Europe and the United States (US) [17], they have recently acquired some attention in Arabic-speaking countries [18]. The studies examining patient preferences in Arabic countries have used the basic methods and general attributes related to health services assessment [13]. Furthermore, older Arabic patients and those with chronic health conditions prefer a paternalistic approach to their care [19]. This may be correlated to physicians’ perception of prioritizing evidence over SDM, sociocultural barriers, and the perception of patients’ unwillingness to take decisions regarding their health [20]. These concepts of patient preferences and SDM are important in countries such as the United Arab Emirates (UAE) where chronic diseases are highly prevalent, and patients are used to paternalistic approach in healthcare [20–22]. Although the UAE government is continuously aiming to improve the healthcare system [23], patient involvement in healthcare remains limited [24].

In OA settings, conjoint analysis (CA), discrete choice experiments (DCEs), best-worst scaling (BWS), and several other methods have been used to elicit patients’ preferences for treatments and understand the trade-offs [8, 25]. Adaptive Choice-Based Conjoint (ACBC) is the most recent and advanced CA technique used to quantify patients’ preferences [26]. The term “Adaptive” refers to the customization of choice tasks to the respondents’ preferred decision criteria [27]. Compared to the conventional methods, the ACBC can assess a large number of attributes (> 5 attributes) [28, 29]. This may help in limiting the extreme response behavior usually encountered in other CA techniques [29]. Additionally, ACBC can also provide information about the second-best options and the unacceptable features of the treatments [29]. However, the ACBC questionnaire takes a longer completion time than the conventional CA as it involves a higher number of attributes and levels [30]. This may cause respondents to be exhausted resulting in incomplete data [31]. Yet, better participants’ engagement while completing the ACBC questionnaire ensures a more accurate prediction of respondents’ choices behavior and may counter the longer completion time [32, 33]. Furthermore, the use of advanced methods such as ACBC can empower patients to discuss their preferences and needs with their physicians, thereby contributing to improved patient-centred care [34].

Despite its potential benefits, the utilization of ACBC in healthcare studies has been limited, especially in OA treatment preferences. Most studies that used ACBC in this context were conducted in the United Kingdom (UK) by Al-Omari and colleagues [26, 35–37], and have been performed in the English language. Al-Omari and colleagues reported that patients’ preferences for OA treatment are driven by avoidance of side effects [26, 36]. They also reported that ACBC is a feasible and useful tool to elicit patients’ preferences for OA treatment [36, 37]. However, one of the main limitations of their studies was related to the small sample size (*n* = 11–43) [26, 35–37].

To the best of our knowledge, there is no available literature on the use of the ACBC method in languages other than English. In turn, the few preference studies conducted were associated with small sample sizes. In light of these considerations, the aim of this study is to elicit patients’ preferences for pharmacological treatment for OA in a large sample of OA patients using a web-based Arabic version of the ACBC questionnaire. The primary research question asked in this study is “What drives patients’ preferences for pharmacological treatment among Arab OA patients?”. This will help in addressing the gap in the utilization of this method in non-English speaking populations and enhance patient-centred care in the UAE through a direct reflection of patients’ needs and preferences.

## Methods

An Arabic version of the ACBC questionnaire was developed to investigate patients’ preferences when choosing drug treatments for OA. The treatment options were described by a set of attributes, further specified by levels set for each attribute.

### Patients and public involvement

In the previous studies conducted by Al-Omari and colleagues [26, 35], the patient and public involvement (PPI) groups were involved in the design of the ACBC questionnaire and the selection of attributes and levels [26].

Since this study was conducted in the UAE, some modifications to the content of the ACBC questionnaire were made to adjust to the cultural and linguistic differences. In the absence of structured PPI groups in the UAE, the researchers formed a group of twenty OA volunteer participants from the public. The volunteers representing both genders (12 females, 8 males), different age groups (25 to 68 years), educational levels (uneducated to master’s degree) and Arab origins (UAE, Jordan, Lebanon, Syria, and Egypt) completed the ACBC questionnaire. The questionnaire web link was sent by email to each participant followed by another email providing the username and password. For validation purposes, participants were then informed of their individual patient preference results and asked if these results were consistent with their stated preferences. All participants confirmed that the ACBC technique predicted their preferences as they intended when they completed the questionnaire. For standardization purposes, each participant was contacted by phone to provide feedback regarding the content and feasibility of using the questionnaire with Arabic OA patients. Subsequently, a few minor amendments to the language of the content were suggested and integrated into the final version of the ACBC questionnaire.

### Participants and settings

A cross-sectional study was conducted on OA patients recruited from the rheumatology and orthopedics clinics at one of the hospitals in the United Arab Emirates, Abu Dhabi between January and June 2022. Adult patients 18 years of age and older, suffering from joint pain, and having a diagnosis of OA from their physicians were recruited. Patients who have acute or chronic illnesses that may contribute to their joint pain other than OA (e.g., osteoporosis, rheumatoid arthritis, septic arthritis, lupus, bursitis, joint injuries) were excluded from this study.

The traditional sample size calculation methods cannot be applied to CA studies for practical reasons [38, 39]. Therefore, there is still no consensus regarding the appropriate sample size for CA studies. A recent systematic review of CA studies indicated that it depends on factors such as the number of subgroup analyses, scenarios, and conjoint tasks [40]. However, it is suggested that the sample size for a CA study should not be less than 300 for one group analysis [41]. Furthermore, reducing the sampling and measurement error in CA studies could be achieved by designing high-quality conjoint tasks and collecting more data from each respondent [40, 41]. Taking into consideration that this study conducted one sample group analysis, it aimed to recruit a minimum of 300 participants.

### ACBC questionnaire

The ACBC questionnaire was developed using Sawtooth Software Lighthouse Studio version 9.13.1. The first screen introduces the participants to the content of the questionnaire (See supplemental Fig. 1). The next set of screens is related to the participants’ demographics and OA medical history (See supplemental Figs. 2–12). The ACBC task starts after the demographics questions and consists of three sections. The first section is “Build Your Own” which presents a complete list of all the attributes and levels and asks the participants to choose their most preferred level for each attribute (See supplemental Fig. 13). The second section is the “screening section” which consists of multiple scenarios generated by the software and customized based on the participant’s previous answers (See Supplemental Fig. 14) and the ‘must have’ and ‘unacceptable’ questions to customize scenarios matching the individual participant preferences (See supplemental Figs. 15 and 16). The final task consists of choice-based questions where participants can choose their preferred scenario from three sets of scenarios (See supplemental Fig. 17).

Since the Sawtooth software is mainly adapted to the English language, the questionnaire had to be translated into the Arabic language by modifying some of the built-in codes with the assistance of a software developer as recommended by the technical team of Sawtooth Software. Primarily, the questionnaire was developed in English language then translated to Arabic by one of the researchers and reviewed by the lead researcher. The Arabic version of the questionnaire was then reviewed by five native Arabic speakers to confirm the language’s accuracy. The rheumatology and orthopaedics healthcare team also reviewed the Arabic version of the questionnaire before piloting with the PPI.

### Defining attributes and levels

The factors that may directly affect patients’ preferences regarding the pharmaceutical treatment of OA were identified as the attributes and levels. The selection of these attributes and levels was based on a previously published systematic review, comprehensive discussion with the PPI group, a previously conducted ACBC feasibility study, and discussions with the physicians, pharmacists of the rheumatology department as well as research methodologists and PPI coordinators.

The original pilot study conducted in the United Kingdom (UK) by Al-Omari and colleagues included eight attributes and 28 levels and recruited 11 participants [35]. The “treatment benefit” attribute was split into “mobility improvement” and “pain reduction” resulting in a total of nine attributes and 31 levels and recruited 43 participants in the following study [26]. This current study has a total number of 10 attributes and 34 levels after including the ‘cost’ attribute, since the variation in insurance coverage among UAE patients may affect their preferences for treatment. A full list of the attributes and levels is presented in supplemental Fig. 13. Comprehensive details about the criteria for including the attributes and levels are discussed in previously published studies by Al-Omari and colleagues [26, 40, 42].

### Questionnaire validation

For validation purposes, three researchers completed the ACBC questionnaire independently assuming two different characters of participants with extreme preference profiles. At the beginning of the task, investigators were provided with documents explaining the profiles regarding specific pharmacological treatment preferences. The main question this task wanted to address was: Given a clear set of preferences, starting from a different random starting point (which is how the ACBC questionnaire works to get started on each occasion), and with different observers armed with these preferences, would we get the same outcome? The answer to this question was “yes”; the ACBC method produced the same outcome for all researchers independently, which matched the set of preferred levels for the extreme preference profiles.

### Data collection

Posters about the study were distributed at the reception of the rheumatology and orthopedics clinics. Two full-time research associates collected the data in the clinics during the period from January until June 2022. Patients who showed interest in participating in the study were directly assisted by the researchers. An information sheet explaining the aim of the study and a consent form was given to each participant. All participants were allowed to raise any inquiries or concerns related to the study with the researchers. The recruited participants were accompanied to a private room to ensure information privacy and patients’ comfort. Touch screen Microsoft pads were provided to the participants to complete the ACBC questionnaire. Participants were given the opportunity to complete the questionnaire independently or be assisted by the researchers during the waiting time for their clinic visit.

### Data analysis

SPSS (version 22.0) was used to analyze the descriptive statistics. The categorical variables were presented in frequencies and percentages while the mean and the standard deviation (SD) were used for continuous variables. The lighthouse studio built-in Hierarchical Bayesian (HB) analysis was used to estimate the relative importance of the attributes and the part-worth (utilities) of the levels. Utilities are estimated through the maximum likelihood of each level [43, 44]. The ACBC HB estimate individual-level utility coefficients and aggregate preference distribution with individual choices by repeating the estimation and borrowing estimates of population-level means and covariance [41, 45–48]. This estimation assumes that respondents answer using an additive process consistent with the multinomial logit rule [43, 44, 49]. The level with the highest utility is the most favourable. The actual utility value given to each level is arbitrarily assigned and the levels’ utilities in each attribute are summed to zero. Therefore, the utility numbers represent the order of the levels and do not have a specific quantitative interpretation. Furthermore, the utility value of one level cannot be compared with the value of another level in another attribute because the ACBC utilities are measured on an interval, rather than a ratio, scale. Therefore, the Z score was used to standardize utility values across all attributes. The relative importance of the attributes is ratio-scaled and relative. The sums of the relative importance of all attributes add up to 100%. A higher relative importance represents a greater impact on patient’s preferences.

## Results

### Patients’ characteristics

One thousand and thirty OA patients completed the online ACBC questionnaire. Approximately 83% of the participants were females. Approximately 33% and 34% were aged 50–59 and 60–69 years, respectively, and only 0.6% were 20–29 years old. The dominant mother tongue language was Arabic (98.7%) since the majority of the study participants originated from UAE (89.5%). The majority of the participants (75.4%) were either uneducated (35.9%) or had school education (39.5%) and only 1% had a doctoral degree. Approximately 66% of the patients were unemployed whereas the minority were either part-time workers or students (0.6%) (See Table 1).

**Table 1:** Demographics of participants (*n* = 1030)

**Characteristic**	**n**	**%**
**Age groups**		
20–29	6	0.6
30–39	35	3.4
40–49	128	12.4
50–59	340	33.0
60–69	348	33.8
70–79	149	14.5
Over 79	24	2.3
**Country of Origin**		
United Arab Emirates	922	89.5
Yaman	18	1.7
Egypt	17	1.7
Jordan	13	1.3
Oman	9	0.9
Syria	7	0.7
Lebanon	5	0.5
Palestine	5	0.5
Other	34	3.3
**Mother Tongue**		
Arabic	1017	98.7
English	10	1.0
Others	3	0.3
**Gender**		
Male	171	16.6
Female	859	83.4
**Education**		
Doctoral degree	10	1.0
Master’s degree	27	2.6
Bachelor’s degree	157	15.2
Diploma	55	5.3
High school certificate	204	19.8
Middle school	99	9.6
Primary school	104	10.1
Not educated	370	35.9
I prefer not to say	4	0.4
**Employment**		
Employed	133	12.9
Employed part-time	3	0.3
Self-employed	7	0.7
Unemployed	675	65.5
Retired	208	20.2
Student	3	0.3
I prefer not to say	1	0.1

### Patients’ OA-associated characteristics

Largely participants suffer from OA in one type of joint (63.4%) and approximately 10.4% had OA in three or more types of joints. The most affected joint by OA was the knee (83.8%) and the least affected joint was the elbow (3.6%). Most patients reported suffering from OA for less than 5 years (38%), while 26.5% of patients reported suffering from OA for more than 10 years. OA joint pain extremely affected the normal life in 28.4% of the patients, whereas 5% reported a normal life not being affected by joint pain. Most patients (53.1%) selected physiotherapy and exercise as their preferred treatment for OA, while 15.9% selected surgical intervention (See Table 2).

**Table 2:** Participants’ OA-related characteristics (*n* = 1030)

**Characteristic**	**n**	**%**
**Affected Joints**		
Knee	863	83.8
Spine/Back/Neck	214	20.8
Shoulder	182	17.7
Feet	134	13.0
Hands	94	9.1
Hip	91	8.8
Elbow	38	3.6
**Multiple affected Joints**		
7 Joints	17	1.7
6 Joints	2	0.2
5 Joints	7	0.7
4 Joints	14	1.4
3 Joints	67	6.5
2 Joints	270	26.2
1 Joint	653	63.4
**Pain interference with normal life**		
Not at all	56	5.4
A little bit	161	15.6
Moderately	257	25.0
Quite a bit	263	25.5
Extremely	293	28.4
**Preferred Treatment**		
Physiotherapy and exercise	547	53.1
Medications	486	47.2
Joint injections	275	26.7
Surgery	164	15.9
**Years suffering from osteoarthritis**		
Less than 5 years	391	38.0
5–10 years	361	35.0
More than 10 years	273	26.5
I do not know	5	0.5

### The relative importance of the attributes

**Fig. 1:**
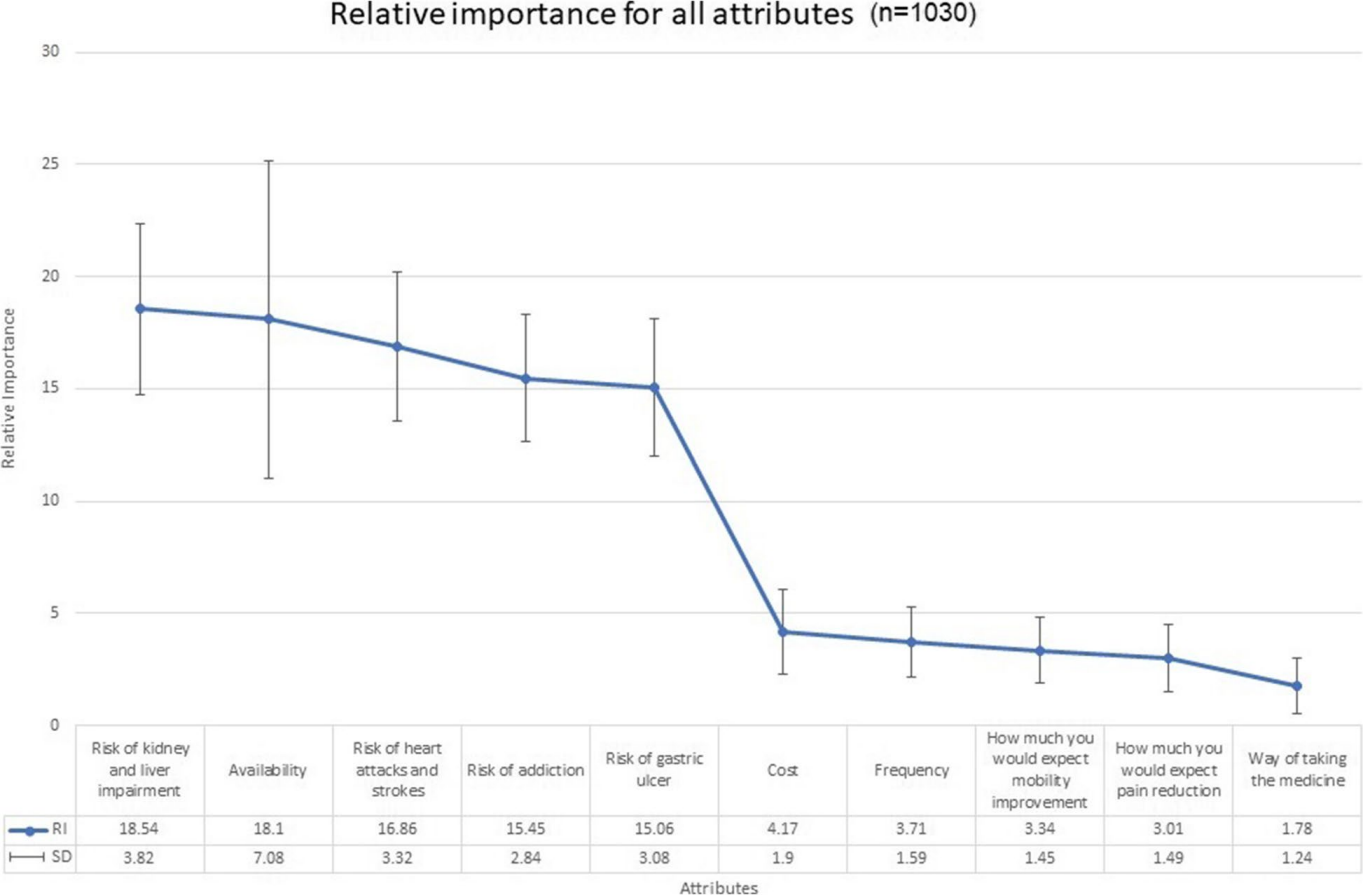
The Relative Importance and Standard Deviation for all attributes

The relative importance (RI) is measured in percentage to determine the relative contribution of different attributes for a given profile. In this case, RI is applied to assess the relative contribution of the attributes for OA treatment. The higher the value of the RI, the more important the attribute. The “Risk of kidney and liver impairment” was the most important attribute (RI = 18.54%), followed by the “Availability” (RI = 18.10%). The least important attribute was the “Way of taking medicine” (RI = 1.78%). The combined RI of the two benefits attributes (mobility improvement and pain reduction) was 6.34% and the combined RI of the four risk attributes (heart attacks and strokes, kidney and liver impairment, addiction, and gastric ulcer) was 65.91%. The highest SD of RI was 7.08 for the “Availability”, whereas the lowest SD was 1.24 for the “Way of taking the medicine” (See Fig. 1). The coefficient of variation (CV) for each attribute was obtained by calculating the ratio of the SD to the mean (represented by the relative importance). The CV provides a better understanding of the relative variability amongst the participant in favouring a particular attribute. The higher the value of CV for an attribute, the greater the variation between responses favouring this attribute. The “Way of taking the medicine” attribute had the highest variation (CV = 0.70) whereas the “Risk of addiction” attribute had the lowest variation (CV = 0.18) (See Table 3).

**Table 3:** Coefficient of variation values of participants’ attributes (*n* = 1030)

**Attribute**	**Coefficient of variation**
Way of taking the medicine	0.70
How much you would expect pain reduction	0.50
Cost	0.46
How much you would expect mobility improvement	0.43
Frequency	0.43
Availability	0.39
Risk of kidney and liver impairment	0.21
Risk of gastric ulcer	0.20
Risk of heart attacks and strokes	0.20
Risk of addiction	0.18

### Utilities (part-worth) of the levels of attributes

**Fig. 2:**
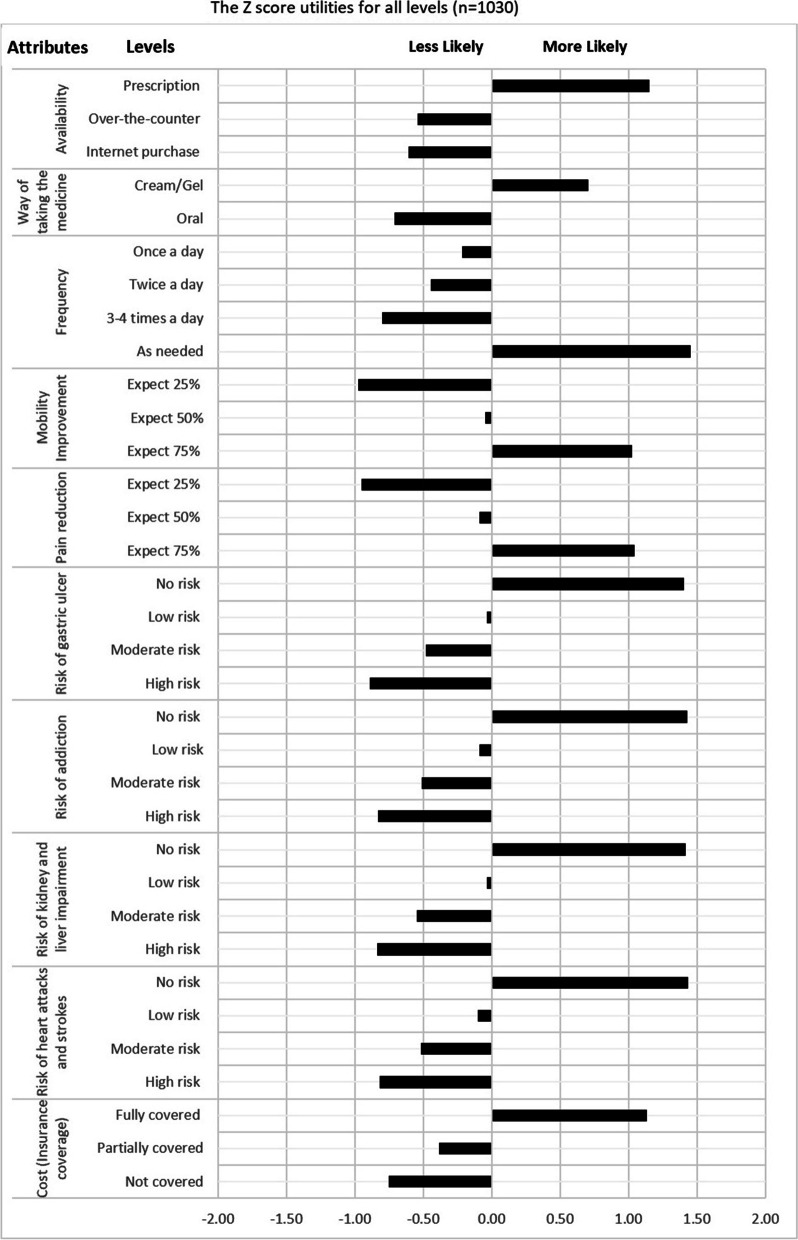
The Z score utilities for all levels

The most preferred scenario for OA treatment for all participants combined was a *prescribed medication applied topically as cream or gel, as needed, provides 75% mobility improvement and 75% pain reduction, has no risk of any of the associated side effects, and is fully covered by the insurance*. The value of the interval utility for each attribute reflects how likely the participants are willing to trade-off a level against another level within the same attribute. The larger value of the utility interval indicates that participants are less likely to trade-off these levels against each other. The easiest levels to trade-off against each other are the levels with the smallest utility interval within each attribute. For example, the availability utilities for “Prescription drug”, “Over-the-counter drug” and “Internet purchase” are 135.9, -64.0, and -71.9, respectively. Therefore, moving from “Prescription drug” to “Over-the-counter drug” and “Over-the-counter drug” to “Internet purchase” would result in interval losses of 199.9 and 7.9. This indicates that it is easier for patients to trade-off over-the-counter with the internet purchase drug than trading-off prescribed medication with any other level. Moreover, the patients’ most preferred level for all risk attributes is the “No” risk. Moving from “No” to “low” toward “moderate” and then “high” risk for all risk attributes, the interval of utilities is gradually reduced. For example, the risk of addiction utility interval between (“No” and “Low”), (“Low” and “Moderate”), and (“Moderate” and “High”) are 107.1, 29.7, and 22.2, respectively. This shows that for the risk of addiction, it is easier for patients to trade-off “High” with “Moderate” risk (utility interval 22.2) than trading off “Low” risk with “No” risk (utility interval 107.2). For the “cost” attribute, a drug “fully covered by the insurance” is the most preferred option for the participants (utility mean 27.5). However, moving from “Fully covered” to “Partially covered” to “Not covered” drugs would result in 36.8 and 8.9 interval losses, respectively. This means that concerning the cost of the OA treatment, patients would probably accept to trade-off between a “Partially covered” and “Not covered” drug (utility mean 8.9) which is not the case if they are asked to trade-off between a “Fully covered” and a “Partially covered” drug (utility mean 36.8) (See Table 4). However, the utility scores for each level are arbitrarily given by the software. Therefore, these scores have been standardized using the z-score to have a mean of 0. Consequently, the utilities of levels across all different attributes would be comparable and potential trade-offs between levels within attributes could be identified. The most preferred level for a selected attribute is reflected through a positive utility score (see Fig. 2).

**Table 4:** Utilities (partworths) for all levels (*n* = 1030)

**Attribute**	**Level**	**Utilities Mean**	**Utility Interval**	**SD**
**Availability**	Prescription drug	135.9		61.5
	Over-the-counter drug	-64.0	199.9	36.1
	Internet purchase drug	-71.9	7.9	32.9
**Way of taking the medicine**	Cream/Gel	1.0		11.1
	Oral	-1.0	2.0	11.1
**Frequency**	As needed	24.7		6.4
	Once a day	-13.5	38.2	8.4
	Twice a day	-7.6	5.9	6.8
	3–4 times a day	-3.6	4	15.2
**Mobility improvement**	Expect 75% mobility improvement	18.5		8.4
	Expect 50% mobility improvement	-0.9	19.4	5.5
	Expect 25% mobility improvement	-17.6	16.7	9.7
**Pain reduction**	Expect 75% pain reduction	16.6		9.6
	Expect 50% pain reduction	-1.4	18	6.1
	Expect 25% pain reduction	-15.1	13.7	9.7
**Risk of gastric ulcer**	No risk of gastric ulcer	93.2		31.4
	Low risk of gastric ulcer	-2.2	95.4	25.6
	Moderate risk of gastric ulcer	-32.0	29.8	22.1
	High risk of gastric ulcer	-59.0	27.0	23.6
**Risk of addiction**	No risk of addiction	100.8	107.2	35.2
	Low risk of addiction	-6.4		26.6
	Moderate risk of addiction	-36.1	29.7	23.5
	High risk of addiction	-58.3	22.2	19.3
**Risk of kidney and liver impairment**	No risk of kidney and liver impairment	98.6		35.4
	Low risk of kidney and liver impairment	-2.4	101.0	22.1
	Moderate risk of kidney and liver impairment	-38.1	35.7	24.0
	High risk of kidney and liver impairment	-58.1	20.0	24.1
**Risk of heart attacks and strokes**	No risk of heart attack and stroke	92.8		35.3
	Low risk of heart attack and stroke	-6.7	99.5	22.6
	Moderate risk of heart attack and stroke	-33.4	26.7	20.9
	High risk of heart attack and stroke	-52.8	19.4	23.1
**Cost**	Fully covered by the insurance	27.5		13.8
	Partially covered by the insurance	-9.3	36.8	6.2
	Not covered by the insurance	-18.2	8.9	9.6

## Discussion

To the best of our knowledge, this is the first application of ACBC in healthcare settings in a language other than English and the first study to elicit patients’ preferences regarding pharmaceutical treatment for OA in the Arab region.

This study recruited 1,030 OA patients suffering from OA in at least one joint. The study investigated their preferences for pharmaceutical treatment using an ACBC questionnaire involving 34 levels of 10 medication attributes. The vast majority of participants were females (83%) and over 50 years of age. This is consistent with previous studies confirming the higher prevalence of OA in females [50–52]. However, 83% is higher than the prevalence reported in previous literature. This may indicate that women are more engaged and interested in participating in this type of study. According to Rigby et al., female patients have a greater preference for acquiring information and understanding medications [53]. Therefore, females are usually more interested than males in physicians’ attention and listening regarding their treatment and health [54, 55]. The older age of the study population may be correlated with the increasing OA cases in patients aged 60 years and above in the Middle East [56–58]. The Prevalence of Rheumatic Diseases and Osteoporosis (PRO) in Dubai study reported that Emirati patients between 41–60 years of age were at increased risk for developing knee OA [59]. Furthermore, the participation of higher older females in our study might be correlated to the severity of OA. A meta-analysis regarding sex differences in OA reported that females ≥ 55 years tended to have more severe knee OA than males [52]. Most of the participants reported that joint pain is extremely affecting their normal life. Yet, participants mainly selected physiotherapy and exercise as their preferred choice of treatment and surgical interventions as the least preferred. Previous studies suggested that physiotherapy and exercise decreased pain, increased health-related quality of life, and delayed surgical interventions [60]. Therefore, this may suggest that OA patients are familiar with OA treatment options and able to make informed decisions about their preferences.

In our study, the combined four side effects attributes accounted for nearly 66% relative importance, while the combined two benefit attributes accounted for just over 6% relative importance. This is consistent with previous research in terms of the importance of side effects attributes preferences for the pharmaceutical treatment of OA [8, 26]. Patients’ preferences and adherence to medications were also seen to be affected by the presence of associated adverse events even if the available drugs were of equal benefit, especially in elderly patients. Precisely, the results of our ACBC study confirm that OA patients’ choice of medication is driven by patients’ avoidance of possible side effects and suggest that there is no difference between Arab OA patients and other ethnic groups in trading-off benefits and side effects. Interestingly, the “Way of taking medicine” attribute was associated with the highest coefficient of variation (0.70) despite its low relative importance (1.78%) compared to all other attributes. Therefore, the dispersion of participants’ preferences regarding the way of taking the medicine may be related to comorbidities, changes in cognitive, motor, and sensory functions which are highly encountered in older people [61]. This also highlights the importance of understanding patients’ preferences details and not focusing only on the most and least important attributes. Therefore, these findings support the crucial value of matching patients’ preferences to treatment recommendations [62]. For example, increased satisfaction with treatment and health-related quality of life have been encountered when patients’ preferences for treatment attributes were incorporated [63]. In turn, a mismatch between physicians’ and patients’ preferences may result in patients’ dissatisfaction, which may impact negatively on patients’ adherence to the recommended treatments [64]. However, the four side effects attributes had a closely similar coefficient of variation (ranging between 0.18 and 0.21). This indicates that OA patients are consistently prioritizing the avoidance of medications’ side effects.

Surprisingly, the “Availability” attribute accounted for 18.10% relative importance (the second most important of all ten attributes). This is much higher than previously reported in the UK by Al-Omari and colleagues (11.6–12.7%) [26], indicating that patients preferred “prescribed medication” more in the UAE than in the UK. This could be due to the addition of the “Cost” attribute in this study which may have made OA patients consider the “Availability” attribute more often, especially since the “Cost” attribute was the sixth most important after the availability and side effects. The availability and cost are interlinked, as the patients will not be able to have the cost covered by the insurance if it is not prescribed by the physician. Furthermore, the use of prescription drugs was seen to be correlated with the patients’ level of education where self-medication was more encountered in younger educated individuals rather than elderly ones [65]. This finding may be extrapolated in our study since most patients were uneducated or had received a limited education. Frequency and way of taking the medication are as important as mobility improvement and pain reduction; these four attributes combined accounted for 11.84% relative importance. Our findings endorse the importance of availability attribute alongside the side effects to patients making decisions about their OA medication options.

In practice, it has been hypothesized that improved medications’ adherence may have a greater influence on the population’s health in comparison to improvements in medical therapies [66]. Several studies in chronic conditions have also correlated poor adherence to an increased risk of hospitalization and overall costs [67]. At an individual level, assessing OA patients’ concerns about treatments, and involving them in treatment decision-making may improve medication adherence and effectiveness [40]. Therefore, improving the understanding of patients’ preferences for OA treatment is of great importance [68]. This explains the necessity of patients’ preferences about treatments to make final treatment decisions [69]. The use of ACBC helps in determining patients’ preferences, allowing healthcare professionals to better understand individual patients’ needs and tailor treatment plans accordingly. This may be particularly significant in Arab countries such as UAE, which have sufficient resources to provide a clinical care model similar to the Western countries [70].

Despite its strengths, this study has some limitations. Anticipating that ACBC is potentially a complicated task for some participants with a limited educational level and computer literacy, the participants were supported by the research associates while completing the ACBC questionnaire. The results may differ if the ACBC questionnaire was completed by the participants without support, as the presence of the researcher may have encouraged participants to complete the task. Despite the large number of recruited participants, our study was conducted in a single healthcare centre, which may limit the generalizability of the findings. Another limitation is that the data related to the associative correlation between patients’ comorbidities other than OA and patients’ preferences to take or avoid was not collected. Therefore, some of the patient’s preferences may not be entirely related to OA. Futuristic studies are needed to clarify the directions of these associations. However, the ACBC questionnaire focused on treatment related to joint pain caused by OA. Moreover, the number of patients approached and those who rejected to participate could not be established. Therefore, we were not able to evaluate the response rate.

## Conclusion

This study marks a significant step forward in the application of an Arabic version of the ACBC questionnaire as a preference-based research method involving patients in healthcare decision-making in the UAE. Arab OA patients are similar to other ethnic groups in trading-off benefits and side effects and consistently prioritizing the avoidance of medications’ side effects. Although the “Way of taking medicine” was the least important attribute it was associated with the highest variation amongst patients. OA patients also prefer prescribed medications to internet-purchased and over-the-counter options.

### Supplementary Information

**Additional file 1: Supplemental figure 1.** An example of the first screen of the ACBC questionnaire (The first screen of the ACBC questionnaire briefly introduces the participants to the content of the questionnaire. This introductory part helps the participants to know what to expect and what kind of questions and tasks are requested from them throughout the whole ACBC questionnaire). **Supplemental figure 2.** An example of patients’ demographics question (age). **Supplemental figure 3.** An example of patients’ demographics question (gender). **Supplemental figure 4.** An example of patients’ demographics question (country of origin). **Supplemental figure 5.** An example of patients’ demographics question (native language). **Supplemental figure 6.** An example of patients’ demographics question (education). **Supplemental figure 7.** An example of patients’ demographics question (employment). **Supplemental figure 8.** An example of OA medical history question (duration of OA). **Supplemental figure 9.** An example of OA medical history question (level of pain). **Supplemental figure 10.** An example of OA medical history question (site of OA). **Supplemental figure 11.** An example of OA medical history question (preferred treatment). **Supplemental figure 12.** An example of OA medical history question (current treatment). **Supplemental figure 13.** An example of the Build Your Own (BYO) questions (In the BYO section, participants can choose their preferred treatment characteristics through the listed attributes and their levels). **Supplemental figure 14.** An example of the screening questions (The screening section presents multiple scenarios for participants. For each scenario, participants are asked to select if the offered treatment characteristics are a “Possibility” or “Not a possibility’ to consider). **Supplemental figure 15.** An example of the “must have” questions (The “Must have” questions allow participants to specify the most important characteristic that needs to be always present in their treatment scenario). **Supplemental figure 16.** An example of the “Unacceptable” questions (The “Unacceptable” questions allow participants to specify the least important characteristic which is not considered if present in their treatment scenario). **Supplemental figure 17.** An example of the choice-task questions. The choice-task questions allow participants to choose their most preferred treatment option among multiple scenarios.

## Data Availability

The datasets generated and/or analysed during the current study are not publicly available due to further ongoing structured research projects. However, data are available from the corresponding author on reasonable request.

## Abbreviations

ACBC: Adaptive Choice-Based Conjoint
BWS: Best-Worst Scaling
CV: Coefficient of Variation
CA: Conjoint analysis
DCEs: Discrete Choice Experiments
HB: Hierarchical Bayesian
OA: Osteoarthritis
PPI: Patient and Public Involvement
RI: Relative Importance
SD: Standard Deviation
UAE: United Arab Emirates
UK: United Kingdom
US: United States
