# Supplementary material for: Exploring patient treatment decision making for osteoarthritis in the UAE: a cross-sectional adaptive choice-based conjoint study
Source: BMC Public Health. 2023 Aug 12;23:1542. doi: 10.1186/s12889-023-16490-1 (PMC10423421; doi:10.1186/s12889-023-16490-1)
Supplement: Supplementary file 1 — Additional file 1: Supplemental figure 1. An example of the first screen of the ACBC questionnaire (The first screen of the ACBC questionnaire briefly introduces the participants to the content of the questionnaire. This introductory part helps the participants to know what to expect and what kind of questions and tasks are requested from them throughout the whole ACBC questionnaire). Supplemental figure 2. An example of patients’ demographics question (age). Supplemental figure 3. An example of patients’ demographics question (gender). Supplemental figure 4. An example of patients’ demographics question (country of origin). Supplemental figure 5. An example of patients’ demographics question (native language). Supplemental figure 6. An example of patients’ demographics question (education). Supplemental figure 7. An example of patients’ demographics question (employment). Supplemental figure 8. An example of OA medical history question (duration of OA). Supplemental figure 9. An example of OA medical history question (level of pain). Supplemental figure 10. An example of OA medical history question (site of OA). Supplemental figure 11. An example of OA medical history question (preferred treatment). Supplemental figure 12. An example of OA medical history question (current treatment). Supplemental figure 13. An example of the Build Your Own (BYO) questions (In the BYO section, participants can choose their preferred treatment characteristics through the listed attributes and their levels). Supplemental figure 14. An example of the screening questions (The screening section presents multiple scenarios for participants. For each scenario, participants are asked to select if the offered treatment characteristics are a “Possibility” or “Not a possibility’ to consider). Supplemental figure 15. An example of the “must have” questions (The “Must have” questions allow participants to specify the most important characteristic that needs to be always present in their treatme [file 12889_2023_16490_MOESM1_ESM.pdf]

Supplemental Material

English Version

Your views on Osteoarthritis Treatment

Welcome to our survey about your preferred choice of medication for your Osteoarthritis.

We would like to thank you for taking part in this study and start by briefly describing the survey.

This survey will be asking you to complete series of questions.
1. The first set of questions will ask you general information about yourself and illness.
2. The second set will ask you to indicate your medication preferences.
3. Then the screening questions will present 4 different medications and ask you if you would consider taking them or not.
4. Then you will be asked to make choices between 3 different medications.

You might feel that the survey seems complicated to start with. However, we are hoping that when you see that the software is listening to your answers and helping you make decisions, you will enjoy the experience.

When you are selecting your answers, please consider that your choices will represent your preferred medication for your chronic illness.

We would like to assure you that all the information you provide in this survey is confidential and will only be used for the purpose of this study.

Completed surveys will be anonymous and your identity will be protected.

Username

Password

Please confirm that you are happy to take part in this study by typing your username and password and then click "Next" button to start completing the survey.

Arabic Version

اختيار الدواء لمعالجة التهاب المفاصل

مرحبا, نشكر مشاركتك في هذه الدراسة , سوف نطرح عليك مجموعة من الاسئلة

1. المجموعة الأولى من الأسئلة هي معلومات عامة عنك و عن مرضك.

2. المجموعة الثانية من الأسئلة عن مواصفات العلاج المفضل لديك.

3. بعد ذلك سنعطيك اربع خيارات من الادويه لتختار المفضل لديك.

4.اخيرا سنطلب منك ان تختار بين ثلاثة انواع من الادويه.

في البدايه يمكن ان تشعر ان الاستبيان معقد و لكننا نأمل انك سوف تستمتع بذلك عندما ترى ان الاستبيان يتأقلم لاجابتك و يساعدك على اتخاذ القرارات.

كل المعلومات في الاستبيان سرية وسوف تستخدم في البحث فقط. هوية المشارك سوف تبقى مجهولة و محميه.

ادخل الاسم المستخدم و كلمة السر و اضغط على "التالي" للبدء بتعبئة الاستبيان.

اسم المستخدم

كلمة السر

الرجاء التأكيد علي موافقتك الدخول في هذا البحث بادخال اسم المستخدم و كلمة السر ثم الضغط على التالي

التالي

Supplemental figure 1. An example of the first screen of the ACBC questionnaire (The first screen of the ACBC questionnaire briefly introduces the participants to the content of the questionnaire. This introductory part helps the participants to know what to expect and what kind of questions and tasks are requested from them throughout the whole ACBC questionnaire).

Supplemental Material

English Version

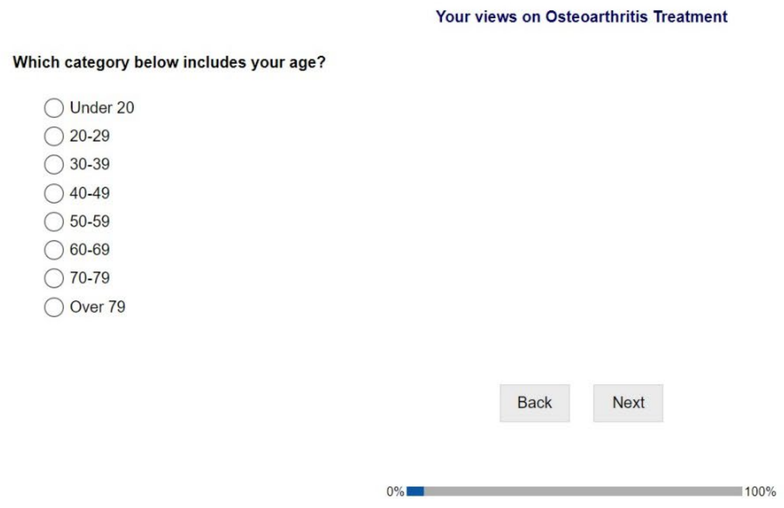

Arabic Version

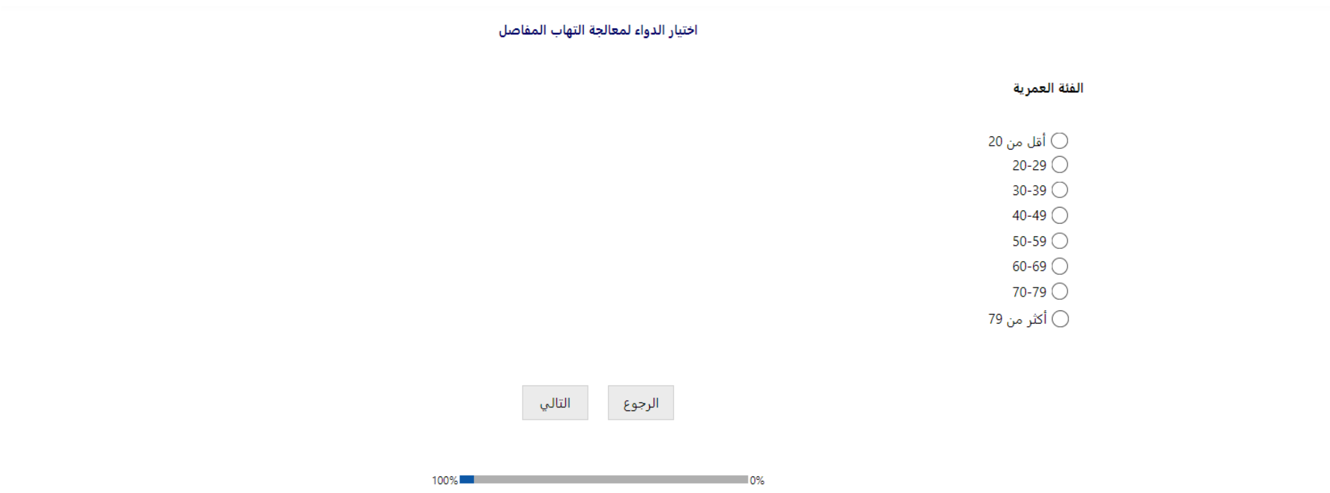

Supplemental figure 2. An example of patients' demographics question (age).

Supplemental Material

English Version

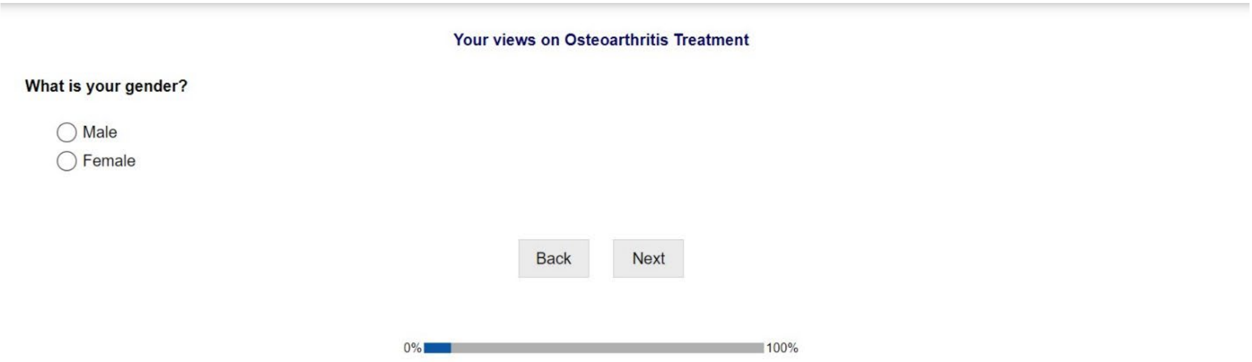

Arabic Version

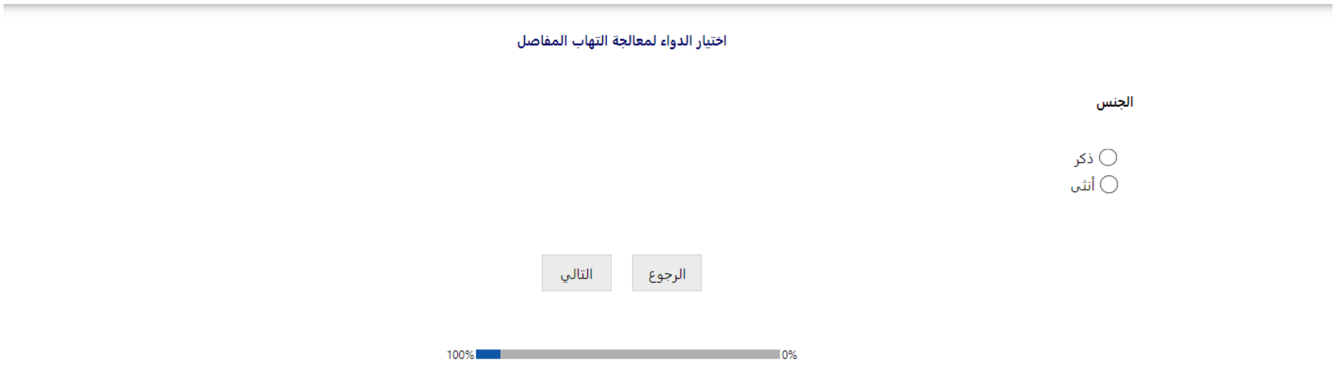

Supplemental figure 3. An example of patients’ demographics question (gender).

Supplemental Material

English Version

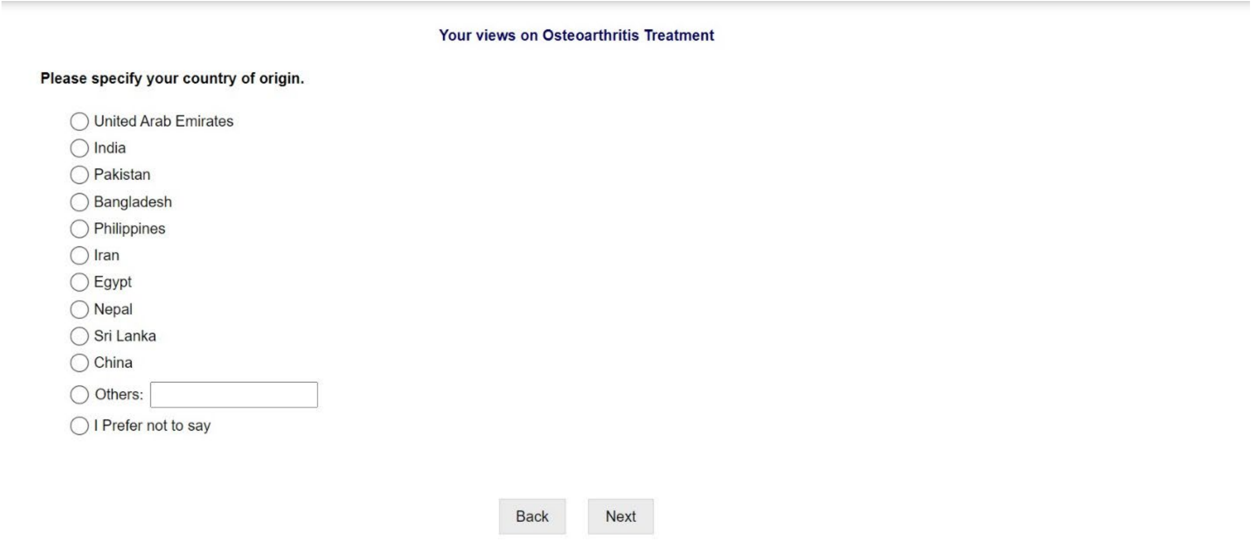

Your views on Osteoarthritis Treatment

Please specify your country of origin.

- ◯ United Arab Emirates
- ◯ India
- ◯ Pakistan
- ◯ Bangladesh
- ◯ Philippines
- ◯ Iran
- ◯ Egypt
- ◯ Nepal
- ◯ Sri Lanka
- ◯ China
- ◯ Others:
- ◯ I Prefer not to say

Back Next

Arabic Version

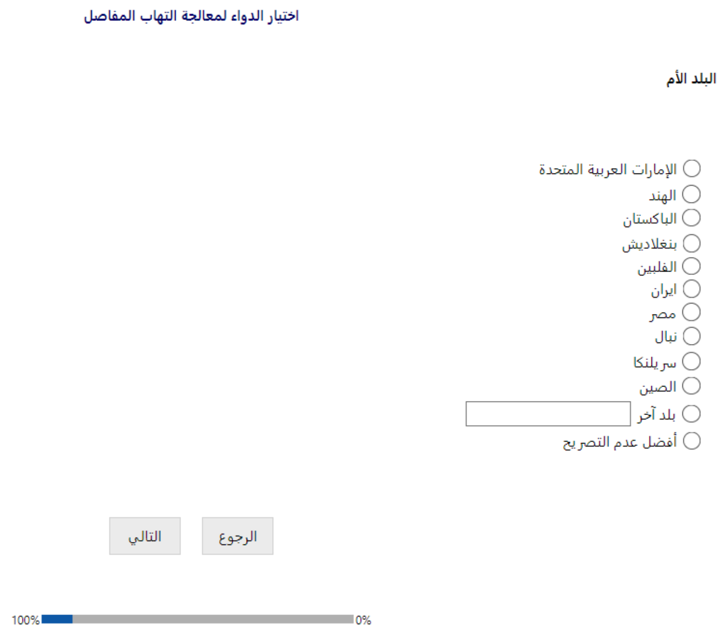

اختيار الدواء لمعالجة التهاب المفاصل

البلد الأم

- ◯ الإمارات العربية المتحدة
- ◯ الهند
- ◯ الباكستان
- ◯ بنغلاديش
- ◯ الفلبين
- ◯ ايران
- ◯ مصر
- ◯ نيبال
- ◯ سريلنكا
- ◯ الصين
- ◯ بلد آخر
- ◯ أفضل عدم التصريح

الرجوع التالي

0% 100%

Supplemental figure 4. An example of patients' demographics question (country of origin).

Supplemental Material

English Version

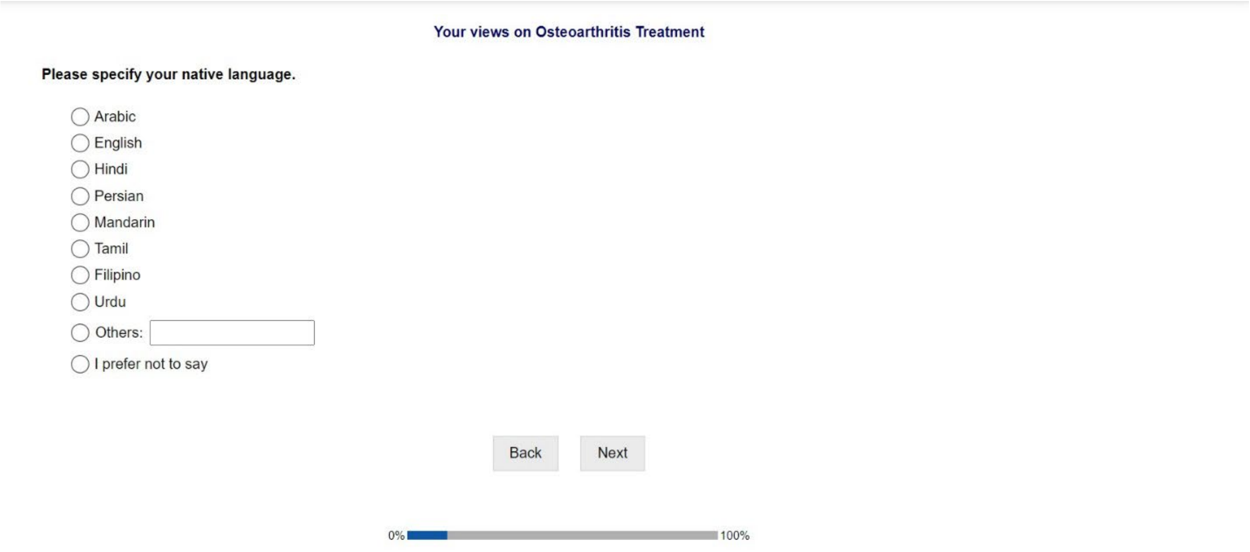

Your views on Osteoarthritis Treatment

Please specify your native language.

- Arabic
- English
- Hindi
- Persian
- Mandarin
- Tamil
- Filipino
- Urdu
- Others:
- I prefer not to say

Back Next

0% 100%

Arabic Version

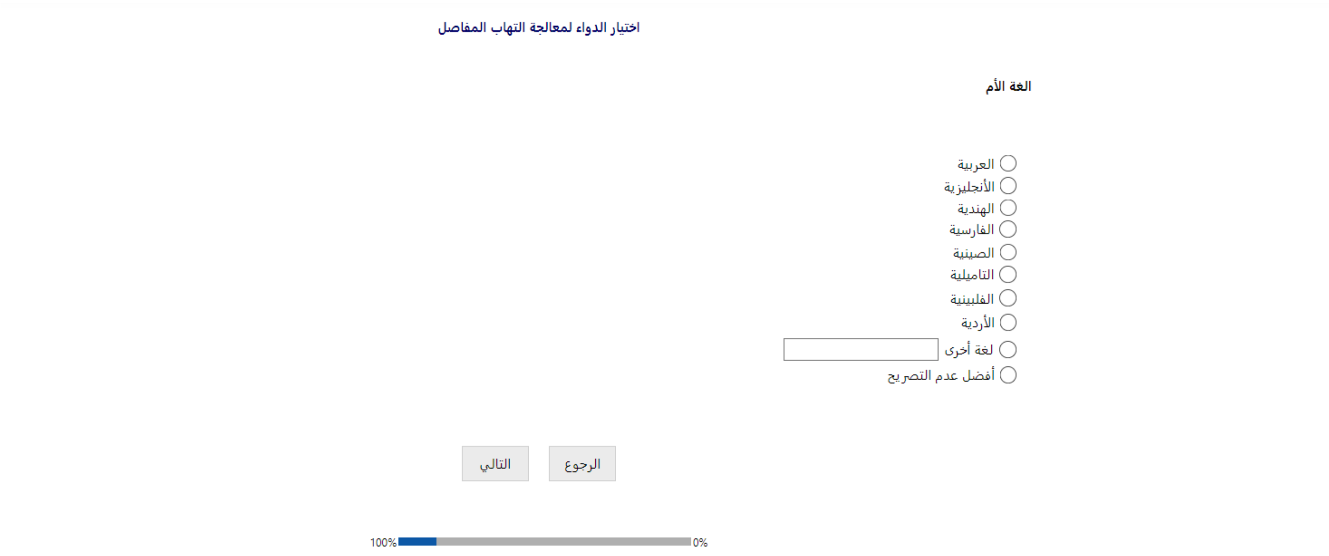

اختيار الدواء لمعالجة التهاب المفاصل

الغة الأم

- العربية
- الأنجليزية
- الهندية
- الفارسية
- الصينية
- التاميلية
- الفلبينية
- الأردية
- لغة أخرى
- أفضل عدم التصريح

التالي الرجوع

100% 0%

Supplemental figure 5. An example of patients' demographics question (native language).

Supplemental Material

English Version

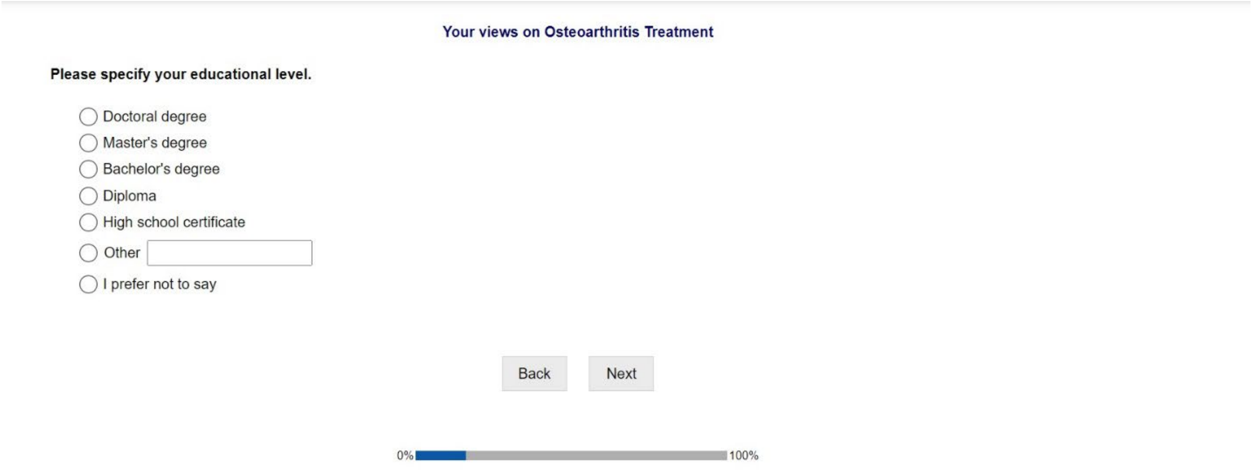
Your views on Osteoarthritis Treatment

Please specify your educational level.

- Doctoral degree
- Master's degree
- Bachelor's degree
- Diploma
- High school certificate
- Other
- I prefer not to say

Back Next

0% 100%

Arabic Version

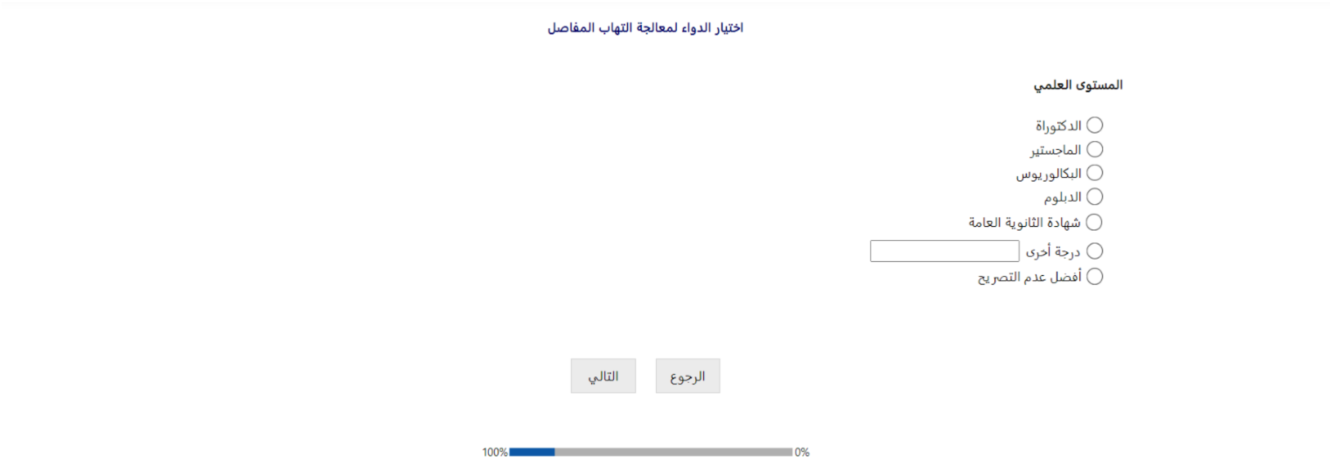
اختيار الدواء لمعالجة التهاب المفاصل

المستوى العلمي

- الدكتوراة
- الماجستير
- البكالوريوس
- الدبلوم
- شهادة الثانوية العامة
- درجة أخرى
- أفضل عدم التصريح

التالي الرجوع

100% 0%

Supplemental figure 6. An example of patients' demographics question (education).

Supplemental Material

English Version

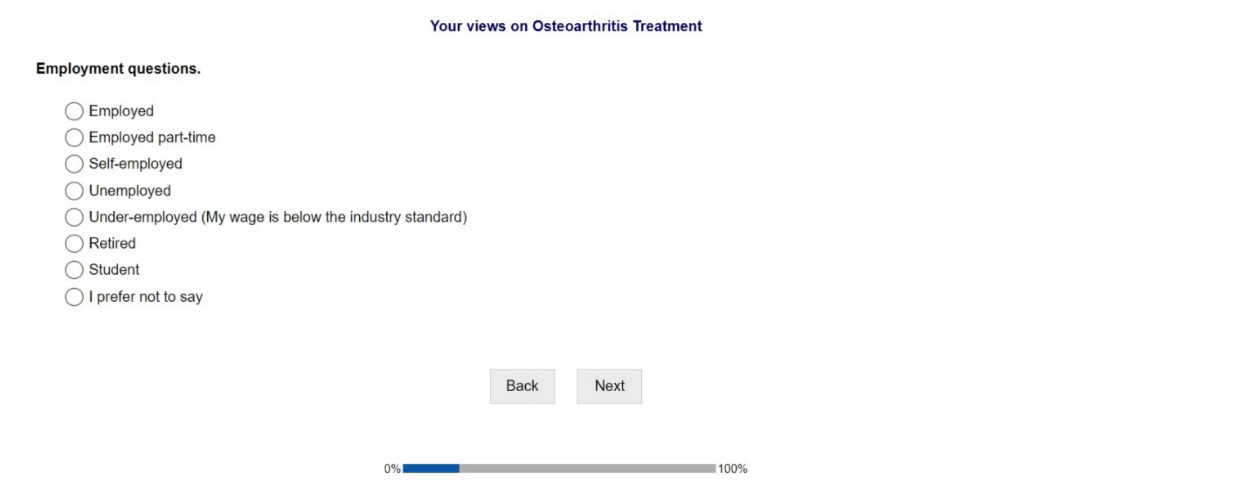
Your views on Osteoarthritis Treatment

Employment questions.

- Employed
- Employed part-time
- Self-employed
- Unemployed
- Under-employed (My wage is below the industry standard)
- Retired
- Student
- I prefer not to say

Back Next

0% 100%

Arabic Version

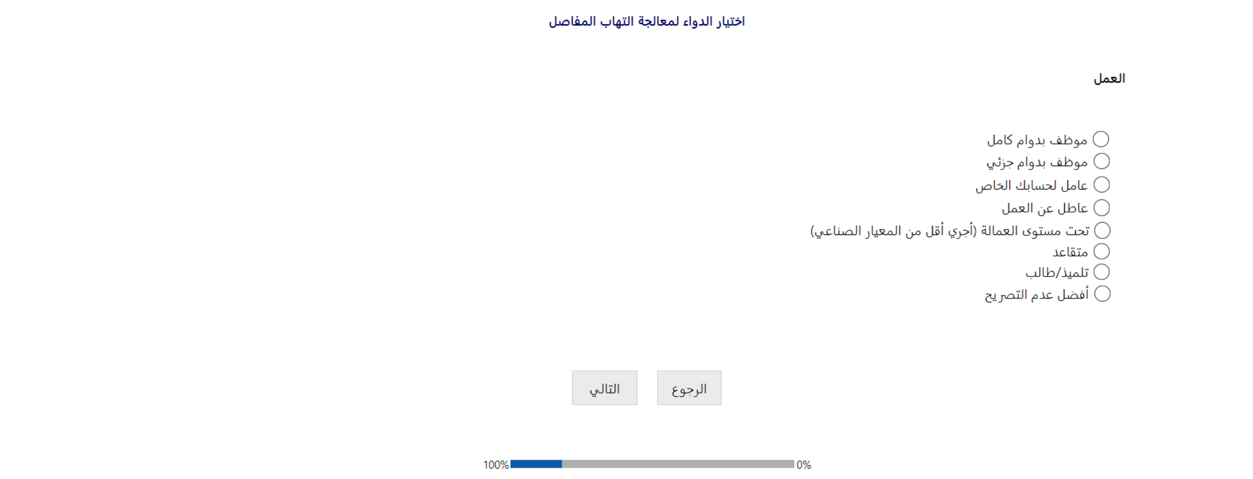
اختيار الدواء لمعالجة التهاب المفاصل

العمل

- موظف بدوام كامل
- موظف بدوام جزئي
- عامل لحسابك الخاص
- عاطل عن العمل
- تحت مستوى العمالة (أجري أقل من المعيار الصناعي)
- متقاعد
- تلميذ/طالب
- أفضل عدم التصريح

التالي الرجوع

100% 0%

Supplemental figure 7. An example of patients' demographics question (employment).

Supplemental Material

English Version

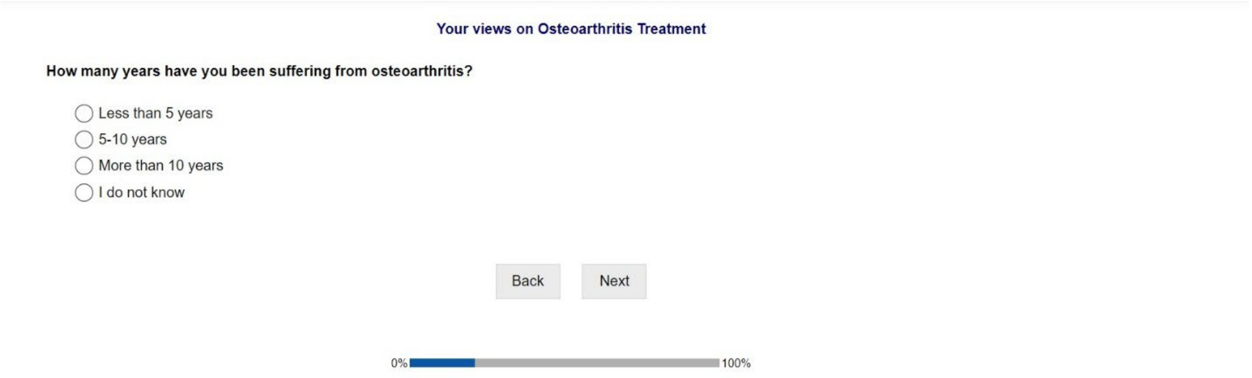

Arabic Version

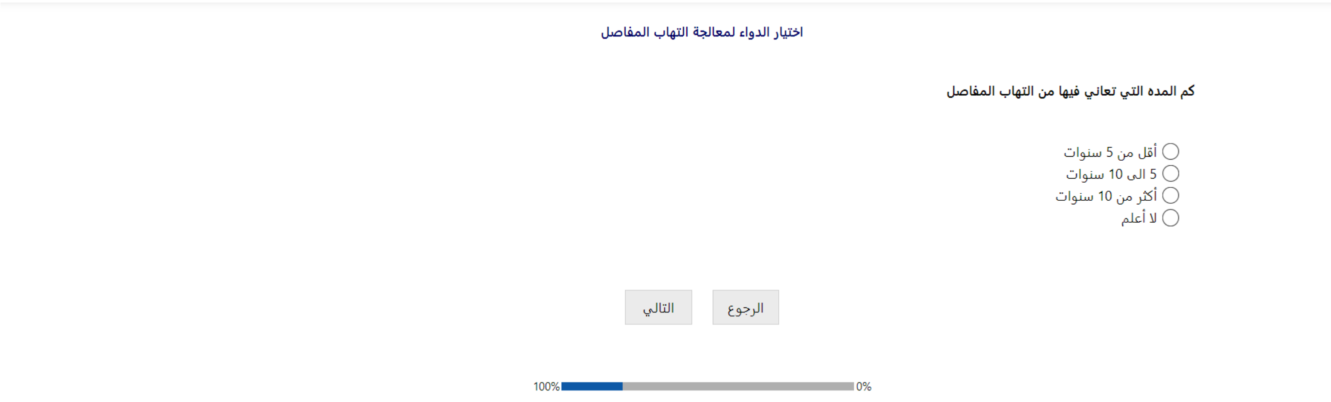

Supplemental figure 8. An example of OA medical history question (duration of OA).

Supplemental Material

English Version

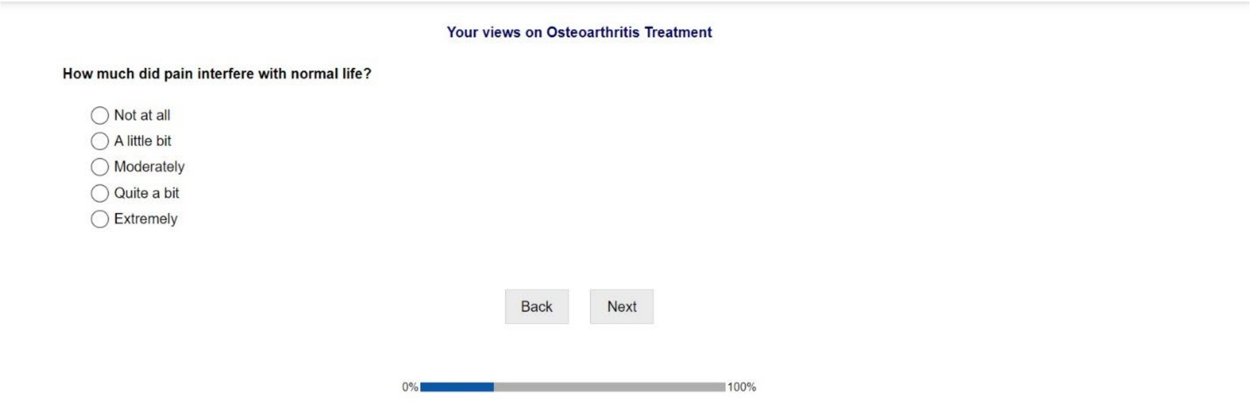

Arabic Version

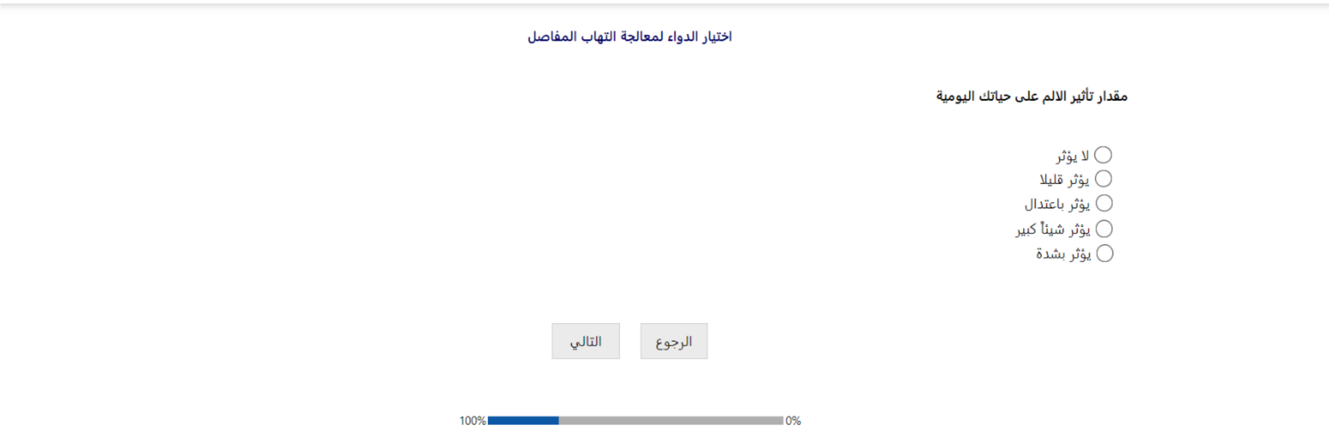

Supplemental figure 9. An example of OA medical history question (level of pain).

Supplemental Material

English Version

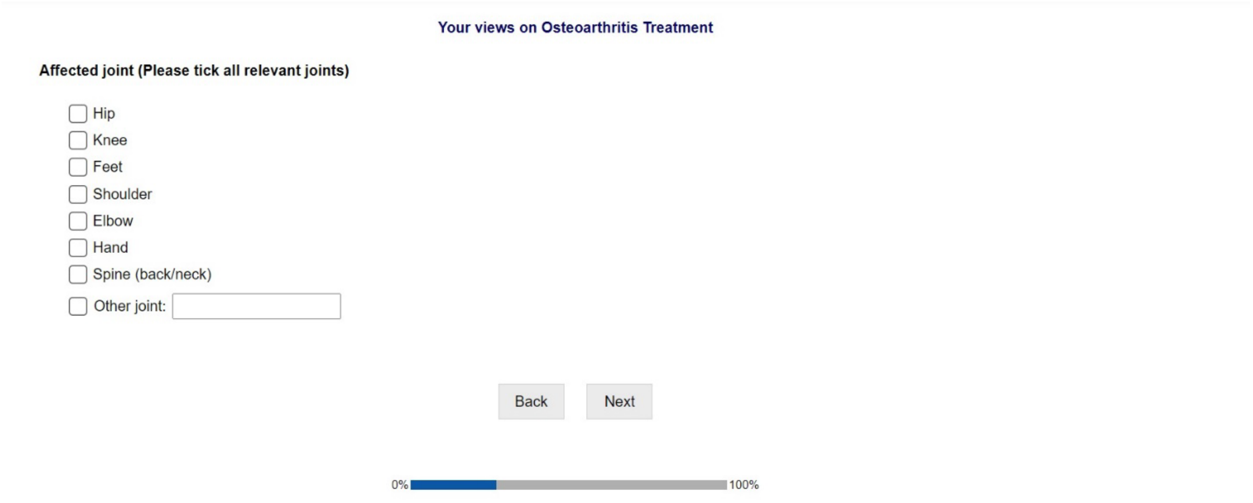

Your views on Osteoarthritis Treatment

Affected joint (Please tick all relevant joints)

- ☐ Hip
- ☐ Knee
- ☐ Feet
- ☐ Shoulder
- ☐ Elbow
- ☐ Hand
- ☐ Spine (back/neck)
- ☐ Other joint:

Back Next

0% 100%

Arabic Version

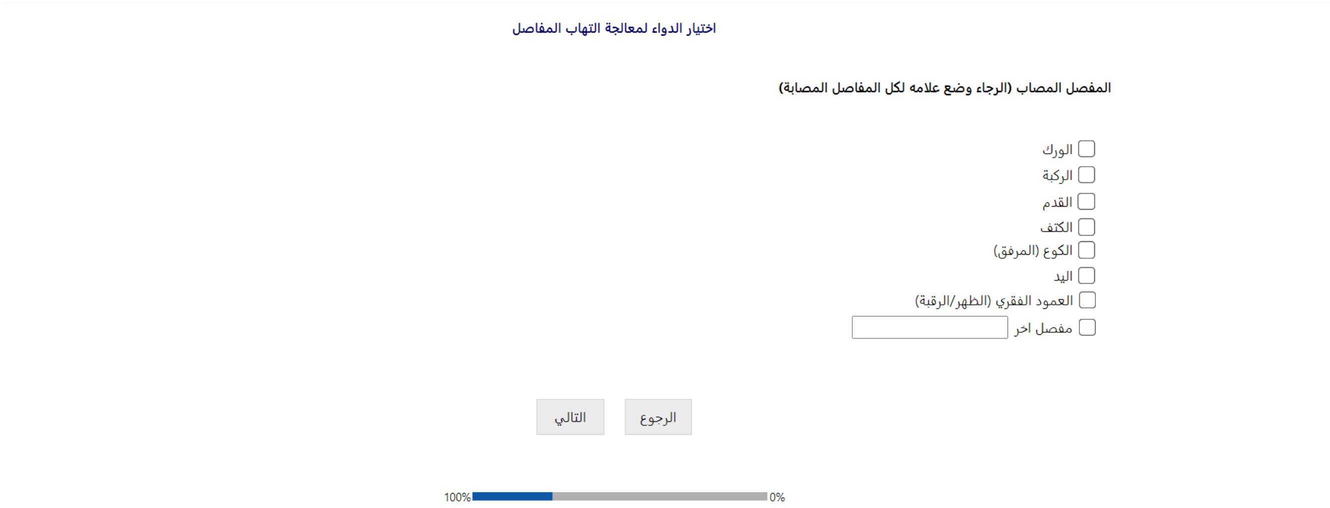

اختيار الدواء لمعالجة التهاب المفاصل

المفصل المصاب (الرجاء وضع علامة لكل المفاصل المصابة)

- ☐ الورك
- ☐ الركبة
- ☐ القدم
- ☐ الكتف
- ☐ الكوع (المرفق)
- ☐ اليد
- ☐ العمود الفقري (الظهر/الرقبة)
- ☐ مفصل اخر

الرجوع التالي

0% 100%

Supplemental figure 10. An example of OA medical history question (site of OA).

Supplemental Material

English Version

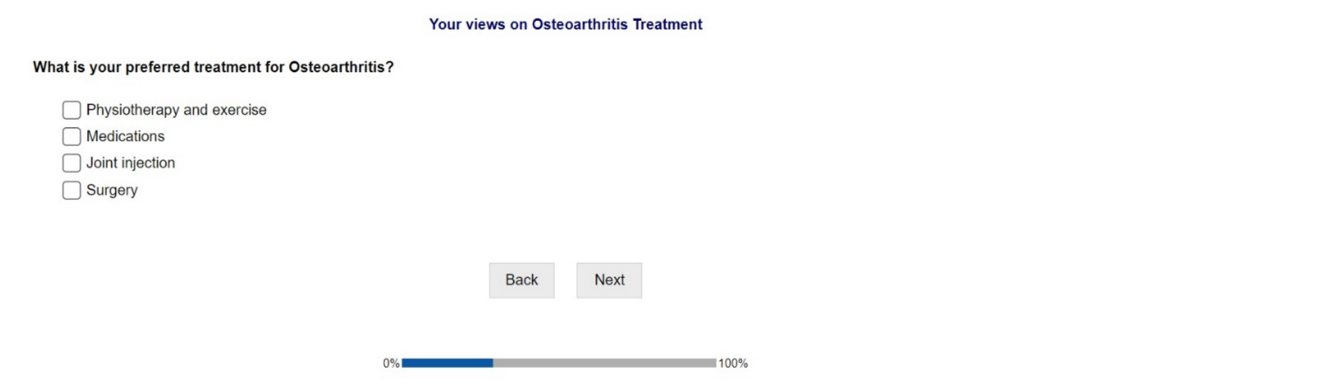

Arabic Version

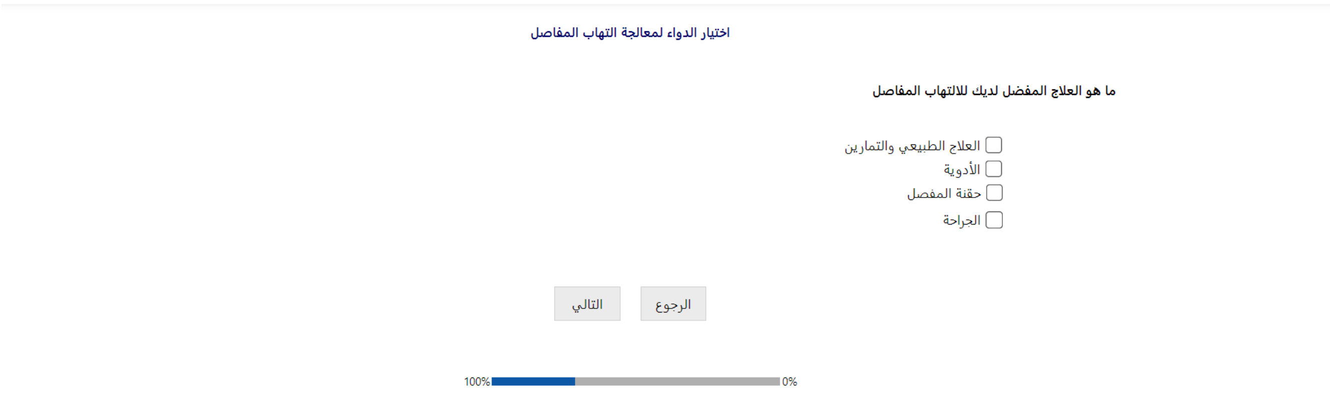

Supplemental figure 11. An example of OA medical history question (preferred treatment).

Supplemental Material

English Version

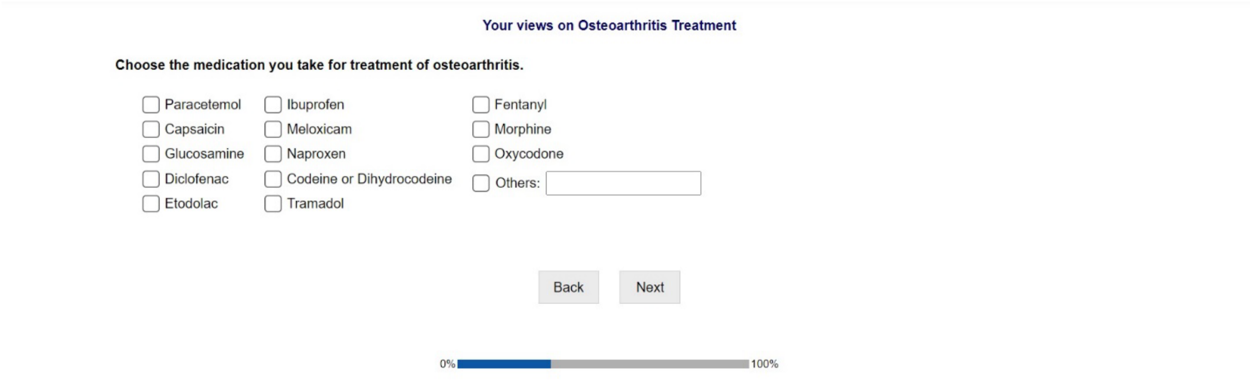

Arabic Version

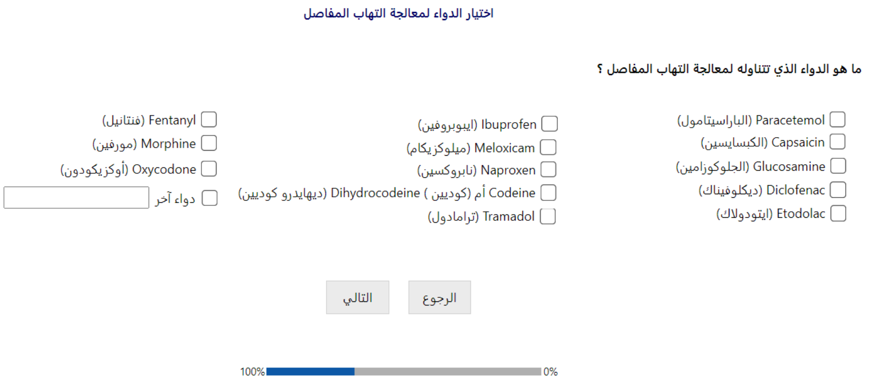

Supplemental figure 12. An example of OA medical history question (current treatment).

Supplemental Material

English Version

Your views on Osteoarthritis Treatment

Please think about the medication you would most like to take for your Osteoarthritis. For each feature, select your preferred level.

Feature	Select Feature
Availability	◯ Prescription drug<br>◯ Over-the-counter drug<br>◯ Internet purchase drug
Way of taking the medicine	◯ Cream/Gel<br>◯ Oral
Frequency	◯ Once a day<br>◯ Twice a day<br>◯ 3-4 times a day<br>◯ As needed
How much you would expect mobility improvement	◯ Expect 25% mobility improvement<br>◯ Expect 50% mobility improvement<br>◯ Expect 75% mobility improvement
How much you would expect pain reduction	◯ Expect 25% pain reduction<br>◯ Expect 50% pain reduction<br>◯ Expect 75% pain reduction
Risk of gastric ulcer	◯ No risk of gastric ulcer<br>◯ Low risk of gastric ulcer<br>◯ Moderate risk of gastric ulcer<br>◯ High risk of gastric ulcer
Risk of addiction	◯ No risk of addiction<br>◯ Low risk of addiction<br>◯ Moderate risk of addiction<br>◯ High risk of addiction
Risk of kidney and liver impairment	◯ No risk of kidney and liver impairment<br>◯ Low risk of kidney and liver impairment<br>◯ Moderate risk of kidney and liver impairment<br>◯ High risk of kidney and liver impairment
Risk of heart attacks and strokes	◯ No risk of heart attacks and strokes<br>◯ Low risk of heart attacks and strokes<br>◯ Moderate risk of heart attacks and strokes<br>◯ High risk of heart attacks and strokes
Cost	◯ Fully covered by the insurance<br>◯ Partially covered by the insurance<br>◯ Not covered by the insurance

Back Next

Supplemental Material

Arabic Version

اختيار الدواء لمعالجة التهاب المفاصل

نرجو ان تفكر في مواصفات الدواء ذو الاحتماليه الاكبر لتتناوله لالتهاب المفاصل. اختر اجابه واحده لكل من المواصفات

الميزة	حدد الميزة
توفر الدواء	بوصفة طبية<br>بدون وصفة طبية<br>يباع من الانترنت
طريقة استخدام الدواء	كريم او هلام<br>عن طريق الفم
وتيرة الاستخدام	مرة في اليوم<br>مرتين في اليوم<br>3-4 مرات في اليوم<br>عند الحاجة فقط
نسبة تحسن الحركة الجسدية المتوقعة	25% تحسن في الحركة الجسدية<br>50% تحسن في الحركة الجسدية<br>75% تحسن في الحركة الجسدية
نسبة تخفيف الألم المتوقعة	25% انخفاض في الألم<br>50% انخفاض في الألم<br>75% انخفاض في الألم
خطر قرحة المعدة	لا يوجد خطر الإصابة بقرحة المعدة<br>خطر ضئيل بالإصابة بقرحة المعدة<br>خطر متوسط بالإصابة بقرحة المعدة<br>خطر مرتفع بالإصابة بقرحة المعدة
خطر الادمان	لا يوجد خطر الإدمان<br>خطر ضئيل للإدمان<br>خطر متوسط للإدمان<br>خطر مرتفع للإدمان
خطر تلف الكلى والكبد	لا يوجد خطر تلف الكلى و الكبد<br>خطر ضئيل لتلف الكلى و الكبد<br>خطر متوسط لتلف الكلى و الكبد<br>خطر مرتفع لتلف الكلى و الكبد
خطر نوبة قلبية وسكتة دماغية	لا يوجد خطر الإصابة بنوبة قلبية و سكتة دماغية<br>خطر ضئيل بالإصابة بنوبة قلبية و سكتة دماغية<br>خطر متوسط بالإصابة بنوبة قلبية و سكتة دماغية<br>خطر مرتفع بالإصابة بنوبة قلبية و سكتة دماغية
التكلفة المادية	تغطية كاملة من جهة التأمين<br>تغطية جزئية من جهة التأمين<br>لا يوجد تغطية من جهة التأمين

التالي   الرجوع

100%   0%

Supplemental figure 13. An example of the Build Your Own (BYO) questions (In the BYO section, participants can choose their preferred treatment characteristics through the listed attributes and their levels).

Supplemental Material

English Version

Your views on Osteoarthritis Treatment

Here, each vertical column represents a medication. For **EACH ONE**, please indicate if you would consider taking this medication or not by ticking the relevant box at the bottom of the column. (2 of 8)

Prescription drug	Prescription drug	Prescription drug	Internet purchase drug
Cream/Gel	Cream/Gel	Oral	Oral
Once a day	Once a day	3-4 times a day	Once a day
Expect 50% mobility improvement	Expect 25% mobility improvement	Expect 25% mobility improvement	Expect 25% mobility improvement
Expect 25% pain reduction	Expect 50% pain reduction	Expect 25% pain reduction	Expect 25% pain reduction
No risk of gastric ulcer	High risk of gastric ulcer	No risk of gastric ulcer	No risk of gastric ulcer
Moderate risk of addiction	No risk of addiction	Low risk of addiction	No risk of addiction
Low risk of kidney and liver impairment	No risk of kidney and liver impairment	No risk of kidney and liver impairment	Low risk of kidney and liver impairment
No risk of heart attacks and strokes	Moderate risk of heart attacks and strokes	Low risk of heart attacks and strokes	No risk of heart attacks and strokes
Partially covered by the insurance	Fully covered by the insurance	Fully covered by the insurance	Fully covered by the insurance
○ A possibility<br>○ Won't work for me	○ A possibility<br>○ Won't work for me	○ A possibility<br>○ Won't work for me	○ A possibility<br>○ Won't work for me

Back Next

Arabic Version

اختيار الدواء لمعالجة التهاب المفاصل

كل عمود يمثل مواصفات دواء. نرجو ان تختار اذا كنت ستوافق في تناول هذا الدواء أم لا

(1 من 8)

بدون وصفة طبية	بوصفة طبية	بوصفة طبية	بدون وصفة طبية
كريم او هلام	كريم او هلام	كريم او هلام	عن طريق الفم
3-4 مرات في اليوم	مرة في اليوم	مرة في اليوم	عند الحاجة فقط
25% تحسن في الحركه الجسدية	25% تحسن في الحركه الجسدية	50% تحسن في الحركه الجسدية	25% تحسن في الحركه الجسدية
25% انخفاض في الألم	75% انخفاض في الألم	25% انخفاض في الألم	25% انخفاض في الألم
لا يوجد خطر الإصابة بقرحة المعدة	خطر مرتفع بالإصابة بقرحة المعدة	خطر ضئيل بالإصابة بقرحة المعدة	لا يوجد خطر الإصابة بقرحة المعدة
خطر ضئيل للإدمان	لا يوجد خطر الإدمان	خطر متوسط للإدمان	لا يوجد خطر الإدمان
لا يوجد خطر تلف الكلى و الكبد	خطر مرتفع لتلف الكلى و الكبد	لا يوجد خطر تلف الكلى و الكبد	لا يوجد خطر تلف الكلى و الكبد
لا يوجد خطر الإصابة بنوبة قلبية و سكتة دماغية	خطر مرتفع بالإصابة بنوبة قلبية و سكتة دماغية	لا يوجد خطر الإصابة بنوبة قلبية و سكتة دماغية	لا يوجد خطر الإصابة بنوبة قلبية و سكتة دماغية
تغطية جزئية من جهة التأمين	تغطية كاملة من جهة التأمين	تغطية كاملة من جهة التأمين	لا يوجد تغطية من جهة التأمين
○ امكانية الاستخدام<br>○ لن ينفع لي	○ امكانية الاستخدام<br>○ لن ينفع لي	○ امكانية الاستخدام<br>○ لن ينفع لي	○ امكانية الاستخدام<br>○ لن ينفع لي

الرجوع التالي

100% 0%

Supplemental figure 14. An example of the screening questions (The screening section presents multiple scenarios for participants. For each scenario, participants are asked to select if the offered treatment characteristics are a "Possibility" or "Not a possibility' to consider).

Supplemental Material

English Version

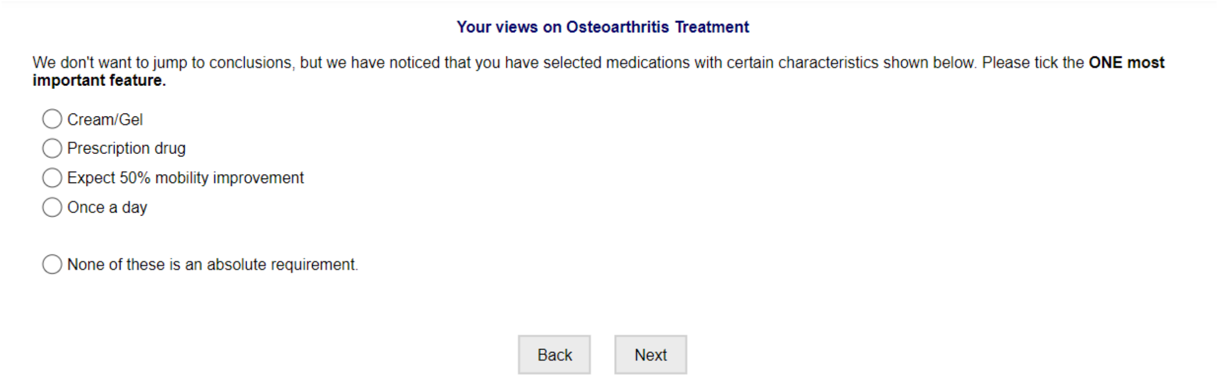

Arabic Version

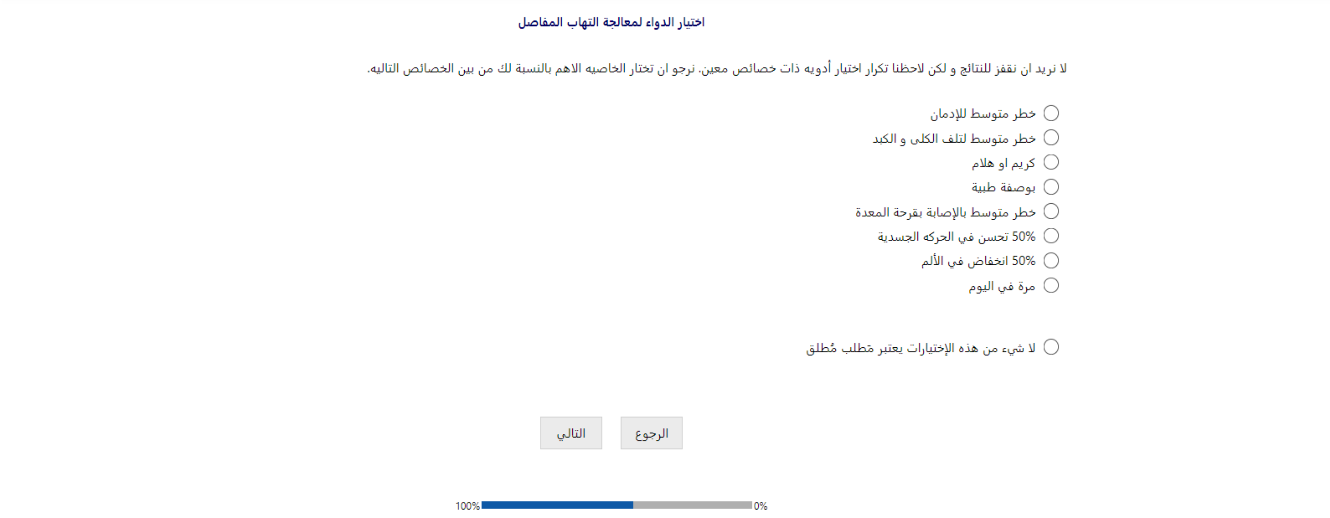

Supplemental figure 15. An example of the "must have" questions (The "Must have" questions allow participants to specify the most important characteristic that needs to be always present in their treatment scenario).

Supplemental Material

English Version

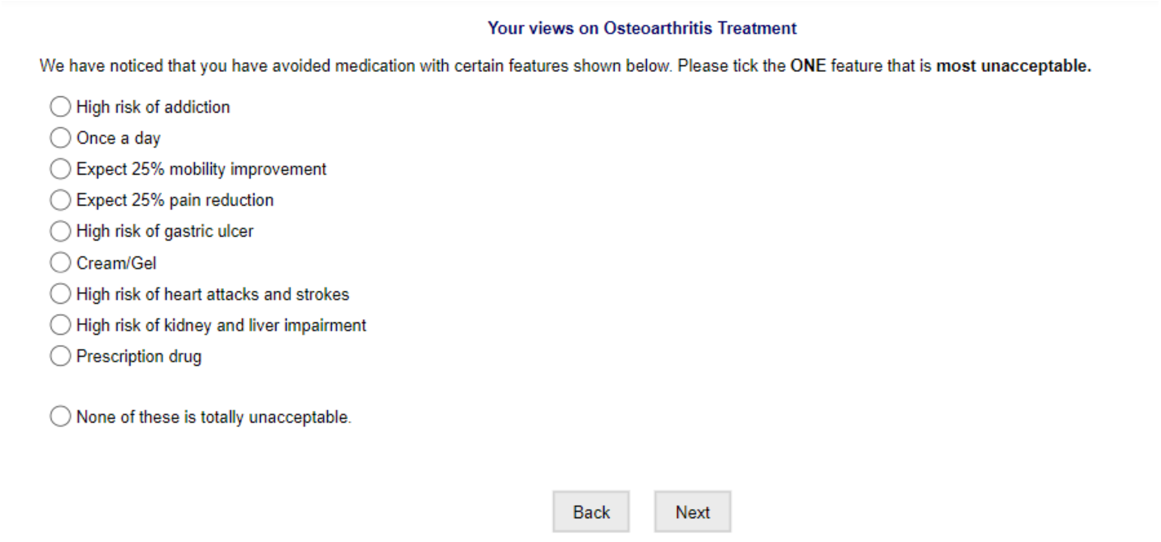
Your views on Osteoarthritis Treatment

We have noticed that you have avoided medication with certain features shown below. Please tick the **ONE** feature that is **most unacceptable.**

- ◯ High risk of addiction
- ◯ Once a day
- ◯ Expect 25% mobility improvement
- ◯ Expect 25% pain reduction
- ◯ High risk of gastric ulcer
- ◯ Cream/Gel
- ◯ High risk of heart attacks and strokes
- ◯ High risk of kidney and liver impairment
- ◯ Prescription drug

- ◯ None of these is totally unacceptable.

Back | Next

Arabic Version

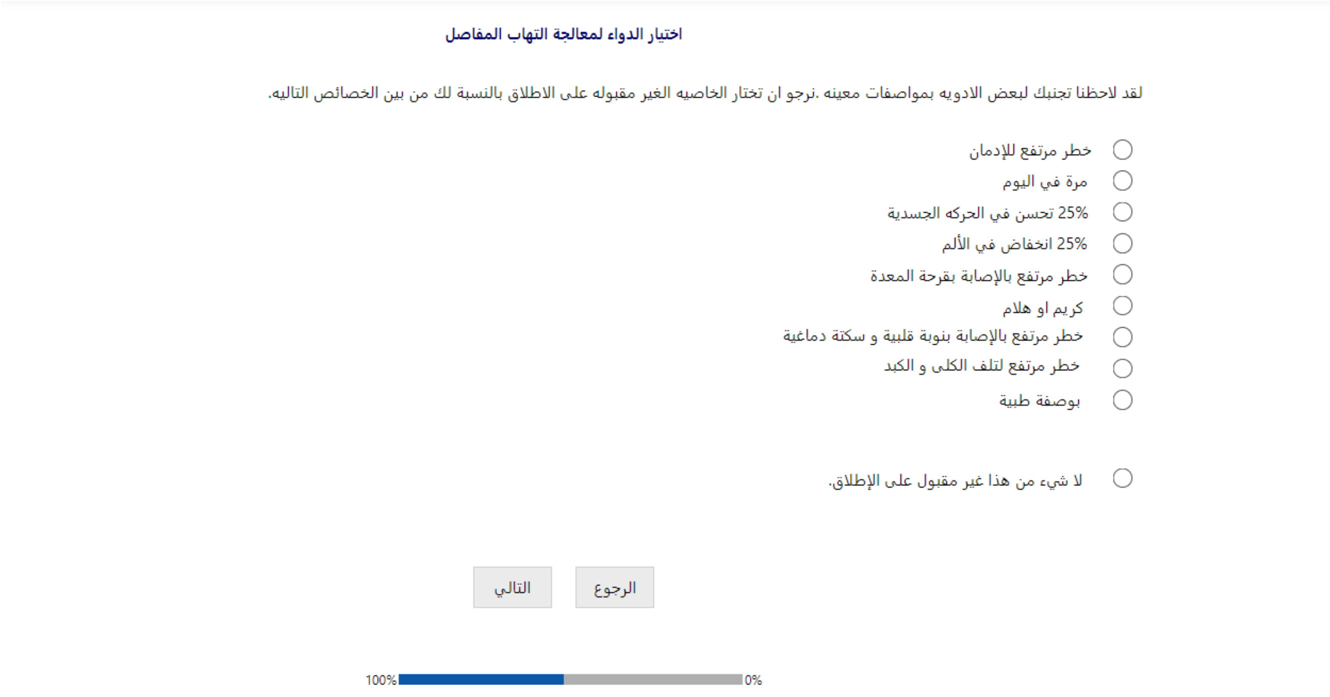
اختيار الدواء لمعالجة التهاب المفاصل

لقد لاحظنا تجنبك لبعض الادويه بمواصفات معينه .نرجو ان تختار الخاصيه الغير مقبوله على الاطلاق بالنسبة لك من بين الخصائص التاليه.

- ◯ خطر مرتفع للإدمان
- ◯ مرة في اليوم
- ◯ 25% تحسن في الحركه الجسدية
- ◯ 25% انخفاض في الألم
- ◯ خطر مرتفع بالإصابة بقرحة المعدة
- ◯ كريم او هلام
- ◯ خطر مرتفع بالإصابة بنوبة قلبية و سكتة دماغية
- ◯ خطر مرتفع لتلف الكلى و الكبد
- ◯ بوصفة طبية

- ◯ لا شيء من هذا غير مقبول على الإطلاق.

الرجوع | التالي

100% | 0%

Supplemental figure 16. An example of the "Unacceptable" questions (The "Unacceptable" questions allow participants to specify the least important characteristic which is not considered if present in their treatment scenario).

Supplemental Material

English Version

Your views on Osteoarthritis Treatment

Here again, each vertical column represents a medication. Among these three, please decide which **ONE** is the best option for you? (Features that are same for each medication are highlighted in grey, so you can focus on the features that are different.) (1 of 5)

Prescription drug	Prescription drug	Prescription drug
Cream/Gel	Cream/Gel	Cream/Gel
Once a day	As needed	Once a day
Expect 25% mobility improvement	Expect 25% mobility improvement	Expect 25% mobility improvement
Expect 25% pain reduction	Expect 25% pain reduction	Expect 25% pain reduction
Low risk of gastric ulcer	No risk of gastric ulcer	Low risk of gastric ulcer
Low risk of addiction	No risk of addiction	No risk of addiction
Low risk of kidney and liver impairment	No risk of kidney and liver impairment	No risk of kidney and liver impairment
No risk of heart attacks and strokes	High risk of heart attacks and strokes	No risk of heart attacks and strokes
Fully covered by the insurance	Fully covered by the insurance	Partially covered by the insurance
○	○	○

Back   Next

Arabic Version

اختيار الدواء لمعالجة التهاب المفاصل

هنا ايضا كل عمود يمثل مواصفات دواء. من هذه الثلاثه نرجو اختيار الدواء المفضل لديك. المواصفات المضلله هي ثابته لكل دواء. لقد ضللناها لتسهل عملية تركيزك على باقي المواصفات.

(1 من 5)

بوصفة طبية	بوصفة طبية	بوصفة طبية
كريم او هلام	كريم او هلام	كريم او هلام
مرة في اليوم	عند الحاجة فقط	مرة في اليوم
25% تحسن في الحركه الجسدية	25% تحسن في الحركه الجسدية	25% تحسن في الحركه الجسدية
25% انخفاض في الألم	25% انخفاض في الألم	25% انخفاض في الألم
خطر ضئيل بالإصابة بقرحة المعدة	لا يوجد خطر الإصابة بقرحة المعدة	خطر ضئيل بالإصابة بقرحة المعدة
لا يوجد خطر الإدمان	لا يوجد خطر الإدمان	خطر ضئيل للإدمان
لا يوجد خطر تلف الكلى و الكبد	لا يوجد خطر تلف الكلى و الكبد	خطر ضئيل لتلف الكلى و الكبد
لا يوجد خطر الإصابة بنوبة قلبية و سكتة دماغية	خطر مرتفع بالإصابة بنوبة قلبية و سكتة دماغية	لا يوجد خطر الإصابة بنوبة قلبية و سكتة دماغية
تغطية جزئية من جهة التأمين	تغطية كاملة من جهة التأمين	تغطية كاملة من جهة التأمين
○	○	○

التالي   الرجوع

100% 0%

Supplemental figure 17. An example of the choice-task questions. The choice-task questions allow participants to choose their most preferred treatment option among multiple scenarios.

Supplemental Material
